# Tracing selection signatures in the pig genome gives evidence for selective pressures on a unique curly hair phenotype in Mangalitza

**DOI:** 10.1038/s41598-020-79037-z

**Published:** 2020-12-17

**Authors:** Kathrin Schachler, Ottmar Distl, Julia Metzger

**Affiliations:** 1 Institute of Animal Breeding and Genetics, University of Veterinary Medicine Hannover, 30559 Hannover, Germany; 2 Veterinary Functional Genomics Group, RG Development & Disease, Max Planck Institute for Molecular Genetics, 14195 Berlin, Germany

**Keywords:** Developmental biology, Morphogenesis, Genetics, Genomics, Comparative genomics

## Abstract

Selection for desirable traits and breed-specific phenotypes has left distinctive footprints in the genome of pigs. As representative of a breed with strong selective traces aiming for robustness, health and performance, the Mangalitza pig, a native curly-haired pig breed from Hungary, was investigated in this study. Whole genome sequencing and SNP chip genotyping was performed to detect runs of homozygosity (ROH) in Mangalitza and Mangalitza-crossbreeds. We identified breed specific ROH regions harboring genes associated with the development of the curly hair type and further characteristics of this breed. Further analysis of two matings of Mangalitza with straight-coated pig breeds confirmed an autosomal dominant inheritance of curly hair. Subsequent scanning of the genome for variant effects on this trait revealed two variants potentially affecting hair follicle development and differentiation. Validation in a large sample set as well as in imputed SNP data confirmed these variants to be Mangalitza-specific. Herein, we demonstrated how strong artificial selection has shaped the genome in Mangalitza pigs and left traces in the form of selection signatures. This knowledge on genomic variation promoting unique phenotypes like curly hair provides an important resource for futures studies unraveling genetic effects for special characteristics in livestock.

## Introduction

Intense human-mediated selection has driven the process of domestication and left footprints in the genome of pigs^1^. In that respect, the need for specific phenotypes has led to similar selective pressures on different domesticated breeds, whereas the genes under selection varied significantly among them^2^. Thus, various studies on SNP data have been performed within or across pig breeds in order to determine regions harboring these genes under potential selection for favored traits^3–10^. It was demonstrated that particularly the detection of homozygous stretches along the genome, designated as runs of homozygosity (ROHs), displayed an effective approach to target functionally important regions as well as to elucidate demography of populations and genetic diversity^4,5^. ROH regions (ROHR) were found to give evidence for regions of strong directional selection for biological pathways related to desirable phenotypes like coat color and type, fertility, muscle and fat development, growth and metabolism^8,9,11–14^ significantly characterizing pig breeds.

Among these traits, the development and shape of hair stands out as a particularly differential and breed defining characteristic of high functional importance. It was proposed that hair coat and its shape has a considerable impact on conduction, convection, water vapor diffusion and radiative transfer between skin surface and the external environmental temperature^15^. In particular, a curled hair shape was proposed to exhibit a beneficial property for thermal insulation in cold temperature conditions to retain body heat^16^ as well as for cooling under hot temperature^17^.

In pigs, a curly hair shape is found as breed characteristic in the Mangalitza, a fatty-type, robust and adaptive endangered pig breed from Hungary^18,19^. The special appearance of its hair coat results from the existence of two curled hair types, namely bristles and additionally pronounced wool hair, which can only be found for this degree in wild boars^20,21^. Besides Mangalitza, curly hair exists only in few other pig breeds including the Turopolje pig^22^, Canastrao pig from Brasilia^23^ or the Mexican Cuino pig^24,25^ expressing a curly and/or hairless type there and was in earlier times exhibited in the extinct Lincolnshire curly-coated pig^26^.

The genetic background of the determination patterns for the hair shape, in particular curly hair, has been studied widely in domestic animals like mice, rabbits, cats, dogs, cattle and horses^27–32^, but has not been determined in Mangalitza pig or other pig breeds so far. It is well known that *keratin (KRT)* and *keratin-associated-protein (KRTAP)* genes, as main structural components of hair fiber^33^ are frequently affected by variants associated with curly or woolly hair among humans and mammals^27,30–32,34–38^. Further genes harboring variants for curly hair are *lipase H gene (LIPH), lysophosphatidic acid receptor 6 gene (LPAR6), transcription factor (SP6) and serum- and glucocorticoid-regulated kinase gene (Sgk3)*^28,29,32,39–41^. Furthermore, an influence on hair curvature and follicle formation was suggested to be derived from *epidermal growth factor receptor (EGFR)* and *fibroblast growth factor (FGF)*-related pathways as well as WNT/ß-catenin-signaling pathway^42,43^. Several of these involved genes and mutations were proposed to be under strong selection in domestic animals promoting such a desired and special phenotype^31,32^.

In our study, we aimed at investigating the genome of the Mangalitza as a representative for a European pig breed with a particular and well-defined phenotype. The objective was to demonstrate how strong targeted selection among breeds has influenced the genomic architecture and promoted selection signatures. As a particular example, we investigated the special type of curly hair in Mangalitza contributing to robustness as well as accounting for adaption as a genetic resource with potential economic value for future breeding programs. Information inferred from ROH analysis and variant annotation were used to target variant effects as result of artificial selection within Mangalitza breed.

## Results

### Scan for selection signatures

Whole genome sequencing (WGS) of nine Mangalitza and three Mangalitza F_1_-crossbreeds (individuals of the first filial generation from a Mangalitza boar and an Angeln Saddleback sow) as well as further variant calling collectively with 22 controls resulted in 23,875,519 SNPs for ROH analysis. In total, an average number of 25,790.50 ROHs with a total mean length of runs of 827,472 kb were called. The Mangalitza exhibited an average of 27,522.78 ROHs per individuum with total mean length of 935,739 kb. Genomic inbreeding coefficients (F_ROH_) based on ROH were estimated to be 0.41 in Mangalitza. In control pig breeds, F_ROH_ ranged from 0.24 (Landrace) to 0.47 (Iberian pigs).

Furthermore, ROH detection additionally performed in SNP chip genotyping data (45,285 SNPs) based on 51 individuals including 19 Mangalitza and 15 Mangalitza-crossbreeds resulted in an average number of ROHs of 97.24 with total mean length of 112,667 kb. In Mangalitza, the mean number of ROHs per individuum was 139.26 with a total mean length of 164,599 kb.

Subsequently, in order to identify Mangalitza specific homozygosity regions, ROHs were filtered for common ROH regions (consensus ROHR) found in Mangalitza in WGS as well as SNP chip genotyping data. We identified 25,508 consensus ROHR in WGS data with a total size (sum of total consensus ROHR) of 164,367 kb and with an average size of 6444 kb per ROHR. In SNP chip genotyping data, 38 consensus ROHR were detected exhibiting a total size of 40,773.10 kb and a mean size of 1072.98 kb per consensus ROHR.

**Figure 1:**
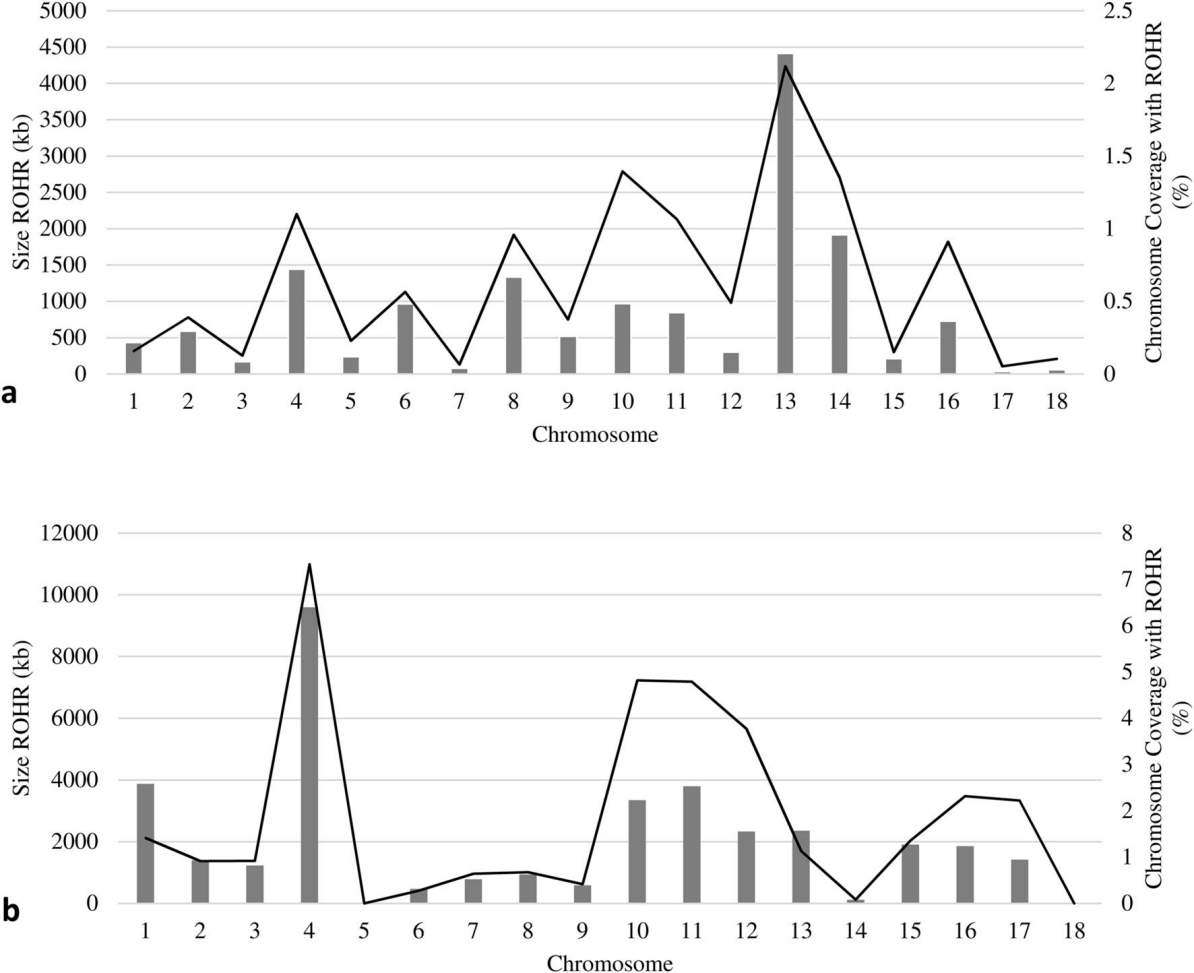
ROH analysis in Mangalitza pigs. Size in kilobasepairs (kb) of consensus ROHR specific for Mangalitza per chromosome (displayed in bars) and their chromosomal coverage in percent (%) (displayed as curve) inferred from WGS data (**a**) and SNP chip genotyping data (**b**).

To narrow down consensus ROHR specific for Mangalitza, we reduced these identified regions for those ROHR absent in Mangalitza-crossbreeds. By this approach, consensus ROHR found in WGS data were reduced to 3401 ROHR with a total size of 15,244 kb and a mean size of 4.48 kb (see Supplementary Table S1). Consensus ROHR from SNP chip genotyping data were only slightly reduced to 36 ROHR with a total length of 35,903.34 kb and mean size of 997.31 kb per ROHR (see Supplementary Table S2). Chromosomal coverage with Mangalitza consensus ROHR was highest on SCA10, 11 and 13 in WGS data and on SCA4, 10 and 11 in SNP chip genotyping data, whereas no consensus ROHR were found on SCA5 as well as on SCA18 (Fig. 1).

### Functional annotation of Mangalitza specific ROHR

Functional annotation of consensus ROHR specific for Mangalitza was carried out to explore biological identities of genes located in potential selection signatures of Mangalitza pigs. Genes detected in WGS data or in SNP chip genotyping data affected a total of 19 biological processes. A high proportion of genes, which primary played a role for cellular (GO:0009987) and metabolic processes (GO:0008152), biological regulation (GO:0065007), cellular component organization or biogenesis (GO:0071840), localization (GO:0051179), response to stimulus (GO:0050896), multicellular organismal (GO:0032501) and developmental processes (GO:0032502) or signaling (GO:0023052), was found. In addition to that, we identified genes enriched in immune system processes (GO:0002376) or in processes affecting behavior (GO:0007610*)* (see Supplementary Table S3). Furthermore, genes located in ROHR found in were involved in a total of 74 different pathways. A large proportion of these genes, which were found in WGS as well as SNP chip genotyping data, affected the inflammation mediated by chemokine and cytokine signaling pathway (P00031), integrin signaling pathway (P00034), PDGF signaling pathway (P00047), Wnt signaling pathway (P00057), EGF receptor signaling pathway (P00018), FGF signaling pathway (P00021) (see Supplementary Table S4). Additionally, a high amount of genes in Mangalitza specific consensus ROHR exclusively found in WGS data was involved in cadherin signaling pathway (P00012), CCKR signaling map (P06959) and heterotrimeric G-protein signaling pathway-Gq alpha and Go alpha mediated pathway (P00027). Moreover, a high proportion of genes detected in ROHR inferred from SNP chip genotyping data contributed to pathways affecting angiogenesis (P00005).

In particular, an accumulation of genes in ROHR involved in pathways related to hair development was detected. Similar findings were made in DAVID functional annotation analysis of WGS data and SNP chip genotyping data, which revealed a high enrichment of genes assigned to the terms coiled coil (P = 1.70E−05 (WGS) and P = 1.8E−02 (SNP chip), e.g. *KRT4, ARAP2*), calcium-activated chloride channel protein (P = 4.60E−02 (WGS), P = 3.30E−04 (SNP chip), e.g. *TCHHL1*)*,* kinesin protein 1B (P = 3.9E−02 (WGS); e.g. *KIF13A*), cadherin and cadherin-like (P = 2.0E−02; 2.1E−02 (WGS), e.g. *CDH10, CDH13*) or S100/CaBP-9k-type calcium binding subdomain (P = 1.10E−04 (SNP chip), e.g. *TCHHL1* among others (see Supplementary Table S5)). Furthermore, we found 12 ROHR located in or close to clusters of genes coding for *KRT* and *KRTAP* on SCA5 at 17.48–18.22 Mb, on SCA12 at 21.01–21.78 Mb and on SCA13 at 193.63–194.51 Mb, at 204.99 Mb and at 207.31–207.40 Mb (see Supplementary Tables S1 and S2), indicating selection signals for characteristic curly hair in Mangalitza.

### Identification of candidate variants for curly hair

**Figure 2:**
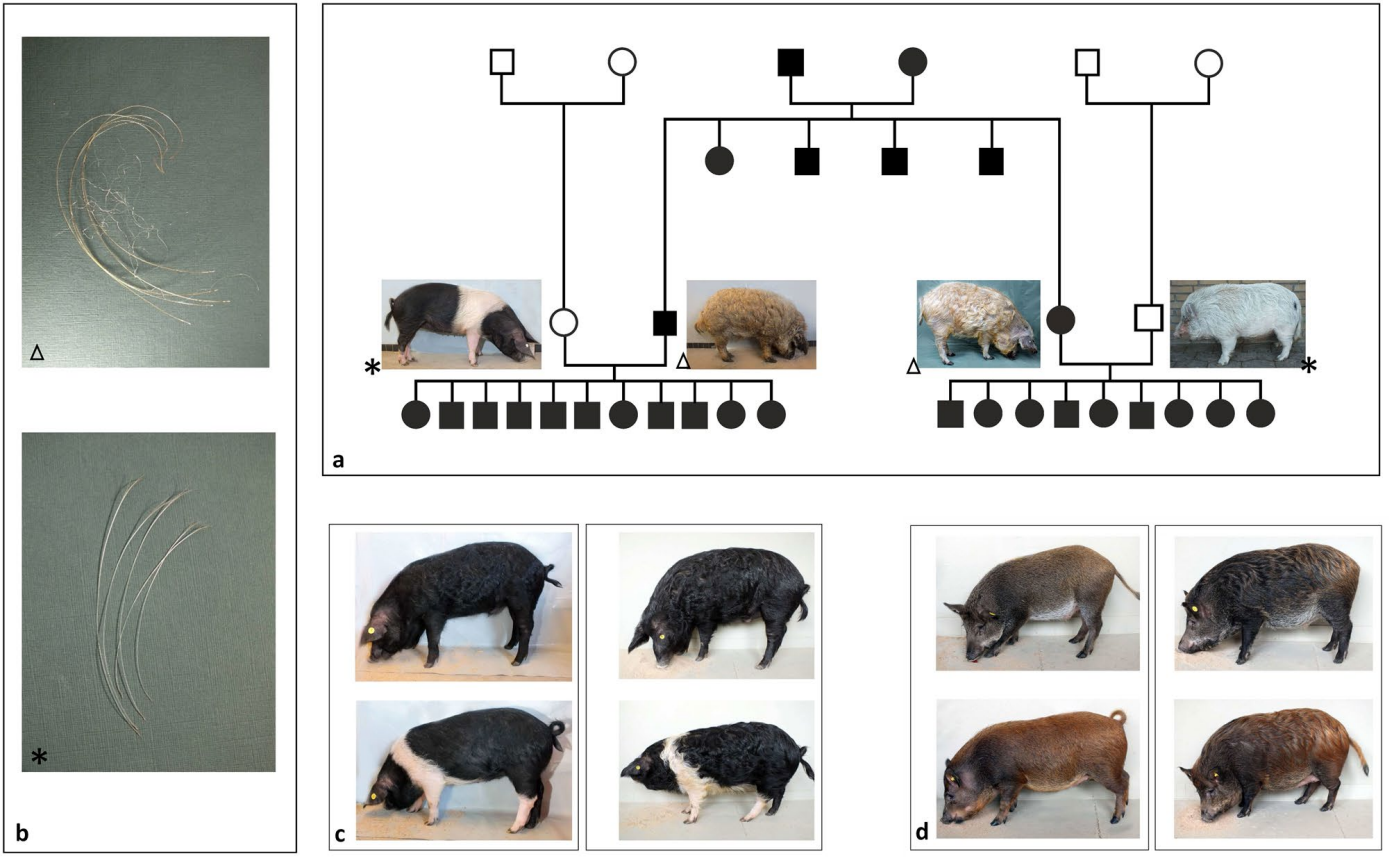
Phenotype in a resource family generated for detailed investigation of curly hair inheritance in Mangalitza. (**a**) Pedigree of resource family: black circle = female, curly-haired; black square = male, curly-haired; white circle = female, straight-haired; white square = male, straight-haired (**b**) Detailed presentation of hair types in curly and straight-haired parenteral generation: the hair in Mangalitza is constituted of curly bristles and of tightly curly wool hair (triangle), whereas in straight-haired breeds bristles are straight (star) (**c**) Development of curly hair in a mating between a Mangalitza boar and an Angeln Saddleback sow at the age of 4 months (left) and 16 months (right) recorded in March. The figure displays one black male (above) and one belted female (below) individual of the litter. At the age of 4 months, the degree of curliness was variable within the litter, but at the age of 16 months, curly coat was completely expressed in all individuals of the litter. (**d**) Development of curly hair in crossbreeds from mating a Mangalitza sow and a miniature pig boar. The figure presents one male (above) and one female (below) individual of the litter. With 5.5 months (September—left) no curly hair was observed within the litter, but at the age of 11 months (March—right), curly coat was expressed all individuals.

To further investigate the genetic background of curly hair in Mangalitza as an example on how selection on special traits impact on the genome of pig breeds, we extended the WGS dataset to in total ten Mangalitza, three Mangalitza-crossbreeds and 56 controls. Furthermore, we studied two resource families produced for targeted investigation of curly hair trait. These families comprised a mating of a curly-haired Mangalitza boar and a straight-haired Angeln Saddleback sow (MAxAS), resulting in a litter of eleven piglets. As well, a curly-haired Mangalitza sow was mated with a straight-haired miniature pig boar (MAxMI), yielding a litter of nine piglets. Both families segregated curly hair in an autosomal dominant manner resulting in curly hair in all 20 progeny (Fig. 2a). Detailed investigation of hair morphology revealed curled bristles and tightly curled wool hair in Mangalitza and straight bristles in Angeln Saddleback and Miniature pig (Fig. 2b). In Mangalitza-crossbreeds the time of manifestation of curly hair varied from 28 days to more than three months (first curly hair were observed at the lateral abdomen) and the degree of curliness appeared to be variable among the individuals (Fig. 2c,d). Finally, all F_1_-piglets developed curly hair and wool hair up to the age of 12 months. Individuals of MAxAS litter (born in November) showed a clear curly hair development at the age of four to five months, whereas all individuals from MAxMI litter (born in April) were straight coated at the same age. During the first year of life of F_1_-generation, we observed the degree of curliness and the occurrence of wool hair to be strongly reduced in summer and the main hair of a straight coated appearance.

A similar segregation was observed in four external breeding and fattening pig farms with distinct filial and backcrossing generations of Mangalitza (n = 42) and straight-haired pig breeds/populations (see Supplementary Table S6). A curly hair was found in all 26 individuals of different age groups belonging to F_1_-generations descending from matings of Mangalitza with straight-haired breeds/populations (Mangalitza × European Wild Boar, Mangalitza × Angeln Saddleback, Mangalitza × Bentheim Black Pied). Furthermore, curly hair was observed in all 12 progeny in a backcrossing to curly-haired Mangalitza ((Mangalitza × European Wild boar) × Mangalitza). However, in one crossing ((Mangalitza × European Wild Boar × Duroc) × Pietrain), a segregation of curly and straight-haired progeny within a litter was observed.

As a next step, we used the variant data set from WGS with 44,785,421 SNPs (7047 with high and 150,520 with moderate effect) and 9,941,843 INDELs (17,894 with high and 6206 with moderate effect) to extract those variants with high or moderate effects exclusively found in a minimum of three Mangalitza and a minimum of one Mangalitza-crossbreed but not in the straight-haired controls. In total, 90 SNPs and 10 INDELs were obtained in this step (see Supplementary Table S7).

### Validation in larger sample sets

Functional annotation of all genes potentially affected by the identified 100 variants revealed twelve coding variants to be predicted to be functionally associated with hair development. Validation in a first sample set of 85 individuals revealed six genetic variants located in *TXLNB* (NC_010443.5:g.25231855G>A—rs336541123), *CYP4F3* (NC_010444.4:g.61866070T>C), *RYBP* (NC_010455.5:g.53728542T>C—rs343014677), *SERPINI1* (NC_010455.5:g.106612073C>G—rs333198858), *TRPM2* (NC_010455.5:g.207222334G>C—rs335212953), *LARP1* (NC_010458.4:g.68450902G>A), which segregated with curly hair phenotype in Mangalitza and did not appear in any other straight-haired pig breed (see Supplementary Table S8). Further genotyping of these six variants in a second larger sample set of 496 individuals exhibited one of these variants, NC_010455.5:g.53728542T>C, to occur in straight-haired individuals, whereas the other five variants were found exclusively in curly-haired Mangalitza and Mangalitza-crossbreeds. (Supplementary Table S9).

To further narrow down variants associated with curly hair development in Mangalitza, we subsequently used a large data set of imputed SNPs based on SNP chip genotyping results from a total of 1275 controls as well as 61 Mangalitza and Mangalitza-crossbreeds. Error rate estimation for imputed data ranged from 0.00 to 0.26 per candidate SNP and mean error rate for five candidate SNPs was 0.11 (see Supplementary Table S10). Genotype frequencies showed, that curly hair associated alleles in NC_010444.4:g.61866070T>C (*CYP4F3*) on SCA2 as well as NC_010455.5:g.207222334G>C (*TRPM2*) on SCA13 were found in a few straight-haired individuals (nine Cinta Senese, one Belorussian Pork Swine, one Nera Siciliana, one Berkshire, two Spotted and one Poland China (USA), one Russian Livenskaya) (Table 1). Screening of further SNP imputed genotypes and alleles revealed additionally one intergenic SNP on SCA2 (NC_010444.4:g.61779350G>T) with few discrepancies in straight-haired individuals close to the variant NC_010444.4:g.61866070T>C (*CYP4F3*) in a high linkage disequilibrium (LD).

**Table 1:** Genotype frequencies of five candidate SNPs for curly validated in imputed data.

Gene	SCA	Position	Genotype	Straight-haired (n = 1275)	Curly-haired (n = 61)
*TXLNB*	1	NC_010443.5: g.25231855G>A	G/G	0.759 (968)	0.344 (21)
			**A/**G	0.191 (244)	0.410 (25)
			**A/A**	0.049 (63)	0.246 (15)
*–*	2	NC_010444.4: g.61779350G>T	G/G	0.998 (1272)	0.115 (7)
			**T**/G	0.002 (3)	0.492 (30)
			**T/T**	0.000 (0)	0.393(24)
*CYP4F3*	2	NC_010444.4: g.61866070T>C	T/T	0.988 (1260)	0.115 (7)
			**C/**T	0.010 (13)	0.492 (30)
			**C/C**	0.002 (2)	0.393 (24)
*SERPINI1*	13	NC_010455.5: g.106612073C>G	C/C	0.919 (1172)	0.148 (9)
			**G/**C	0.073 (93)	0.475 (29)
			**G/G**	0.008 (10)	0.377 (23)
*TRPM2*	13	NC_010455.5: g.207222334G>C	G/G	0.999 (1274)	0.639 (5)
			**C/**G	0.001 (1)	0.279 (17)
			**C/C**	0.000 (0)	0.082 (39)
*LARP1*	16	NC_010458.4: g.68450902G>A	G/G	0.947 (1207)	0.607 (37)
			**A/**G	0.048 (61)	0.328 (20)
			**A/A**	0.005 (7)	0.066 (4)

Curly-associated alleles are printed in bold.

Evaluation of co-segregation of the two coding SNPs on SCA2 and 13 revealed that curly hair phenotype could be explained in all curly-haired individuals except for two Mangalitza and two Mangalitza-crossbreeds descendent from crossing (Mangalitza × European Wild Boar × Duroc) × Pietrain (Table 2). In addition to that, both variants were found to be located close to Mangalitza specific ROHR at SCA2: 56,016,731–56,017,548 and SCA13: 205,652,439–205,652,711 indicating a potential signature of selection in these regions. The *cytochrome P450 4F3 *(*CYP4F3)* variant, resulting in a substitution of histidine to arginine (p.His488Arg) was predicted to be benign (score 0.00), the same as *transient receptor potential cation channel subfamily M member 2 (TRPM2)* variant, a glutamic acid to glutamine (p.Glu110Gln) substitution in both transcripts (benign, score 0.005). However, both variants were found to be located in a highly conserved region.

**Table 2:** Validation of candidate variants for curly hair development in an imputed dataset of a total of 1336 individuals.

	1/1–1/1	1/1–0/1	1/1–0/0	0/1–1/1	0/1–0/1	0/1–0/0	0/0–1/1	0/0–0/1	0/0–0/0
**NC_010444.4:g.61866070T>C/NC_010455.5:g.207222334G>C**									
Straight-haired (1275)	0	0	2	0	0	13	0	1	1260
Curly-haired (61)									
Mangalitza-crossbreed (21)	0	0	0	0	1	18	0	0	2
Mangalitza (40)	3	10	11	2	3	6	0	3	2

The genotypic distribution of combined genotypes of the two investigated variants NC_010444.4:g.61866070T>C *(CYP4F3)* and NC_010455.5:g.207222334G>C *(TRPM2)* detected in 61 curly-haired and 1275 straight-haired pigs is demonstrated by numbers of homozygous wild type (0/0), heterozygous (0/1) and homozygous mutant (1/1) genotypes.

### Evaluation of structural variant involvement

Positional evaluation of structural variants in WGS data found exclusively in all Mangalitza and Mangalitza-crossbreeds but not in straight-haired controls within the range of 40 Mb proximal and distal to the two candidate SNPs did not reveal any results. However, we identified one region on SCA13 at 143,207,109 to 143,457,136 bp, which was predicted to be a tandem duplication resulting in a gene fusion of the two novel genes ENSSSCG00000042345 and ENSSSCG00000045503 (see Supplementary Fig. S1) in nine of ten Mangalitza but not in Mangalitza-crossbreeds and straight-haired controls. Integrative Genomics Viewer (IGV) (https://igv.org/) displayed this variant to be present in all ten Mangalitza as well as Mangalitza-crossbreeds. However, further validation revealed no copy number variation within the predicted region in seven curly and three straight-haired individuals suggesting the occurrence of a translocation instead of a tandem duplication. A gene fusion, as it was predicted, could be confirmed by a PCR-amplicon comprising the gene fusion at suggested breakpoints in these Mangalitza samples but not in straight-haired controls. Sanger sequencing of this PCR product confirmed the gene fusion, but revealed a distal shift of the first predicted breakpoint from 143,207,109 to 143,207,203 bp. Finally, in further testing we found the PCR product to be amplifiable in 163 of 189 Mangalitza samples, but also in ten (European wild boars) of 114 straight-haired controls.

## Discussion

Scanning for ROHs in the genome of Mangalitza has advanced the understanding of the important role of selection signals significantly marked as footprints in the genome of pigs. The high number of ROHs and subsequent high estimated inbreeding coefficient (F_ROH_) of 0.41 is presumably a result from the strong decrease of population size of this by now endangered breed, which was nearly extinct in the nineteen-seventies due to the changing demand in pig production for lean meat and productivity^19^. Previous studies also detected quite low diversity in Mangalitza in comparison to other European as well as Asian pig populations^5,44^. As well, similar (0.415) or lower F_ROH_ (0.09–0.14) were reported in previous study in SNP chip genotyping data of Mangalitza pigs^45,46^. In contrast to that, only Iberian pigs were found with higher F_ROH_ in the breeds investigated in our study.

However, although today not widely used for meat production, Mangalitza exhibit particular features, which make this breed special and of particular interest in genomics. On the one hand, it is known as a fat type pig, producing fine quality meat as a delicacy with a considerably high proportion of intramuscular fat and a high amount of unsaturated fatty acids compared to other pig breeds^47^. In that respect, it was no surprise that we identified several genes in Mangalitza consensus ROHR, which were proposed to have an impact on fat metabolism including *lipase I (LIPI)* and *phospholipase A2 group IID (PLA2G2D)* influencing fatty acid composition of meat^48,49^ or *phospholipase A2 group V* (*PLA2G5), hydroxysteroid 11-beta dehydrogenase 1 (HSD11B1)* and *diacylglycerol kinase delta (DGKD)*, which have a crucial role in insulin resistance and the susceptibility for obesity^50–52^.

In addition to that, the Mangalitza is known as a specifically robust breed due to the protective layer of fat, but also to its generally observed strong disease resistance, strong motherliness and above all, its characteristic curly hair^19^. Thus, interestingly, we noticed that some genes identified in Mangalitza specific ROHR were associated with disease resistance and immune system response as well as with behavioral traits. In addition to that, we detected *leucine rich repeats and calponin homology domain containing 1* (*LRCH1)* within a ROHR associated with leg and body conformation as well as soundness traits in pigs^53,54^.

Above all, we found strong evidence in the genome of Mangalitza pigs for targeted selection for curly hair in this study. Notably, all five clusters of genes coding for hair fiber components *KRT* and *KRTAP*, currently annotated in the reference genome Sscrofa11.1 (https://www.ensembl.org/index.html; https://www.ncbi.nlm.nih.gov/), were found to be within or close to consensus ROHR. Moreover, we identified selection signatures in WNT signaling, *EGFR* and *FGF* signaling pathways, which play a crucial role in hair follicle differentiation and formation^42,43^. Similar to that, other studies on ROH in pigs also revealed hair morphologic traits fixed in potential selection signatures. For example, a previous analysis in Chinese pigs displayed ROH islands in genes encoding *fibroblast growth factor 3* and *4* (*FGF3* and *FGF4*) related to hair ridge^10^.

Thus, targeting curly hair developmental genes and potential causative variants for curly hair in Mangalitza, we found evidence for an autosomal dominant inheritance in our resource families. These results contrast with previous predictions from crossings of Mangalitza with Cornwall, Middle White and Large White, which suggested an intermediary hair type for F_1_-piglets^20,55–57^ with a uniform and non-variable degree of hair curliness. In addition to that, we confirmed the occurrence of wool hair in Mangalitza crossbreeds as found in Berkshire, Cornwall and Lincolnshire x Mangalitza crossbreeds^58^ but rejected in other observational studies^20,57^. We assume that these inconsistent findings are due to varying manifestation ages and different seasonal time points under study in these publications or might be due to differential gene effects. Similar to observations in curly coated horses^59^, we found evidence for an enhanced and accelerated development of curly hair and wool hair in colder seasons in comparison to warmer seasons. The reason for these temperature dependent developmental differences remains unclear, although an influence of slick hair on improved regulation of body temperature in hot climate was suggested^60^.

Scanning of the Mangalitza genomes for causative variants for curly hair rejected the hypothesis of such variants in known candidate genes from other species but instead revealed two breed-specific coding variants in *CYP4F3* and *TRPM2* jointly segregating with the curly hair phenotype in all but four individuals. In both genes we found evidence for their role in skin and hair development and differentiation^61–68^.

*CYP4F3,* encoding a member of the cytochrome P450 enzymes, was found to be expressed in human and rodent skin^62^ as well as in a reconstructed human epidermal model from adult hair follicles^63^. It was proposed, that cytochromes P450 are up-regulated during keratinocyte differentiation and thus play a fundamental role for the development of these cell types, which in turn promote hair follicle morphogenesis^61^. Similar to that, various TRP subfamilies were also identified in epidermal keratinocytes^64–66^ and in the inner root sheet and infundibulum of hair follicles^67^. A wavy hair type with curly whiskers observed in *TRPV3* knockout mice was assumed to be a result from TRP channel dependent regulation of epidermal growth factor receptor signaling orchestrating keratinocyte terminal differentiation^68^. Due to these potential functional effects on hair morphogenesis of the candidate genes, we propose that the two missense variants might play an important role in shaping the Mangalitza pig’s hair into a curly hair type. Particularly strong evidence can be found in our validation on DNA samples, which revealed the two variants to be Mangalitza-specific. This was underlined by the large imputation dataset. Only very few individuals from other breeds with a described straight hair coat were predicted to harbor a mutant allele, which is presumably the result of a false-positive variant calling we could predict according to our evaluation of imputation accuracy based on validated genotypes. Regarding the functional effects of these two variants, we assume that the alteration of the amino acid sequence might affect protein function in these conserved regions despite the prediction of a potential benign effect, similar as it has been found in other studies on curly coated species^32^.

In addition to that, we suggest that more than one variant is causative for the specific hair type of Mangalitza pigs. This evidence is supported by the origin of Mangalitza developed by crossbreeding of Alföldi, Szalonta and Bakony breeds with the Serbian Sumadia pig^19^. Both, the Bakony and Alföldi breed, were curly coated^19^, presumably due to different variant effects. Crossings of these breeds might have resulted in an intermixture of these variants. Similar findings were reported in curly coated horses, whose dominant curly hair phenotype was associated with either *KRT25-* or *SP6*-variant or both^32^. Moreover, in cats, different breed-specific variants have been identified to be causative for curly hair^29,40,69^.

Subsequently, in Mangalitza pigs, we expect an additional variant to play a role in curly hair development, which has not been identified yet. In Sscrofa11.1, in total 110 *KRT* and *KRTAP* genes are annotated, whereas in human reference (GRCh38.p13) 161 *KRTs* and *KRTAP* as well as further 175 pseudogenes can be found (https://www.ncbi.nlm.nih.gov/). It was reported that Ensembl (release 93) harbors half the number of annotated genes in the pig genome to what has been reported for human^70^. Therefore, we suppose that a further curly-associated variant might have stayed undetected due to annotational limitations in the pig genome so far.

In conclusion, the present study demonstrated how strong selection for defined phenotypes has shaped genomic structure in Mangalitza, a native and endangered Hungarian pig breed. Artificial selection left traces in the genome in the form of selection signatures for breed specific features with special regard to curly hair phenotype in pigs. These investigations will support the preservation of unique phenotypes often found in endangered breeds with potential economic value as genetic future resource.

## Methods

### Samples and phenotypes

Hair, blood or tissue samples were collected from 115 Mangalitza, 62 Mangalitza-crossbreeds, 3 Angeln Saddleback (traditional and endangered pig breed, characterized by a black color with a white belt)^71^, 12 Bentheim Black Pied (traditional pig breed from the region of Bentheim in the West of Germany, colored white with black spots)^71^, 16 Husum Red Pied, ten Husum Red Pied-crossbreeds, nine German Landrace-crossbreeds, one Miniature pig, one Iberico, three Kune Kune, one Pietrain and one Pot-bellied pig. All work on animals has been performed following the national and international guidelines for animal welfare. EDTA-blood and hair sampling was approved by the animal ethics committee of the Lower Saxony state veterinary office Landesamt für Verbraucherschutz und Lebensmittelsicherheit, Oldenburg, Germany (registration number 33.9-42502-05-17A217). Furthermore, tissue samples were collected at the slaughterhouse. All individuals were phenotypically characterized for their hair type classified as straight or curly and presence of under wool was also recorded. Further 347 samples, including 107 German Landrace, 108 German Large White, 18 Goettingen Minipig, 30 Leicoma, 11 Mini-Lewe, one Meishan, one Pietrain, one Bentheim Black Pied, one Husum Red Pied, two Mangalitza and 67 European wild boars were obtained from the bio-bank of the Institute of Animal Breeding and Genetics, University of Veterinary Medicine Hanover.

### Whole genome sequencing

WGS was performed in five Mangalitza of which two individuals were also used for the production of the resource family (Mangalitza boar and sow) as well as in samples from three curly-haired Mangalitza × Angeln Saddleback F_1_ progeny and in the straight-haired Angeln Saddlebback sow and the Miniature pig boar used for mating. Genomic DNA of these animals was isolated from of 1000 µl EDTA-blood or 50 hair roots using chloroform extraction^36^. DNA fragmentation was done with M220 focused-ultrasonicator (Covaris, Inc., Woburn, Massachusetts, USA) using the following settings: duty factor 20%, peak incident power 50, 200 cycles per burst for 45 s. Library preparation was performed using the NEBNext Ultra II DNA Library Prep Kit for Illumina (New England Biolabs, Ipswich, Massachusetts, USA) including phosphorylation, dA-tailing, adaptor ligation and size selection using AMPure XP beads (Beckman Coulter GmbH, Brea, California, USA). The quality of the libraries was estimated on an Agilent 2100 Bioanalyzer (Agilent Technologies, Santa Clara, California, USA) using the Agilent High Sensitivity DNA Kit (Agilent). Subsequently, the libraries were adjusted to a concentration of 4 nmol and sequenced paired-end for 2 × 150 bp on an Illumina NextSeq500 (Illumina, San Diego, California, USA). All data were submitted to sequence read archive (SRA, NCBI, see Supplementary Table S11). In addition to that, further fastq-files from five Mangalitza and 54 pigs of 24 breeds, including three Korean wild boars and one wild boar Switzerland as straight-haired control samples were downloaded from the SRA, so that WGS data of a total of 69 pigs were used in our analysis. All fastq-files were quality controlled using fastqc, version 0.11.5^72^ and underwent indexing with Picard tools (http://broadinstitute.github.io/picard/, version 2.9.4). Adapter trimming and low complexity filters were applied using the fastq processing tool fastp, version 0.20.0^73^ (detect_adapter_for_pe, -low_complexity_filter, -complexity_threshold 1, -cut_front -cut_front_window_size 1 -cut_front_mean_quality 20 -cut_tail -cut_tail_window_size 1 -cut_tail_mean_quality 20 -qualified_quality_phred 15 -unqualified_percent_limit 70 -n_base_limit 50 -average_qual 0 -disable_length_filtering -disable_trim_poly_g). Finally, all files were mapped to the reference genome Sscrofa11.1 (ftp://ftp.ensembl.org) using Burrows-Wheeler Alignment tool (BWA), version 0.7.13^74^. Variant data were called using GATK software, version 3.7^75^, tools Base Recalibrator, Haplotype Caller^76^, Base Quality Score Recalibrator (BQSR) and Calculate Genotype Posteriors and underwent variant effect prediction using SNPEff (version 4.3t, build 2017-11-24)^77^. For further analysis of the obtained variants, only sites with mean depth values (over all individuals) greater than or equal to two (-min-meanDP 10) and a maximum read depth of 1000 (-max_depth 1000) were admitted and SCA X, Mt and Un were excluded using VCFtools, version 0.1.16^78^.

### Genotyping on porcine 50 K SNP chip

In addition to WGS, we performed genotyping on the Genomic Profiler Porcine 50 K SNP chip (Illumina), including approximately 51,000 SNPs, for 56 EDTA-blood and hair samples as well as for genomic DNA, isolated from 1 ml EDTA-blood, hair roots and tissue samples using chloroform extraction^36^. These samples included 20 curly-haired Mangalitza, 18 curly-haired Mangalitza-crossbreeds, one straight-haired Mangalitza-crossbreed and 17 individuals from ten straight-haired pig breeds/populations (see Supplementary Table S11). For quality control using PLINK, version 1.90^79^, a genotyping rate of 0.99 and a minor allele frequency of 0.05 was required and sex chromosomes were excluded, resulting in a total of 45,285 SNPs and 55 individuals (37 curly-haired as cases and 18 straight-haired pigs as controls) remaining for further analysis.

### Runs of homozygosity

For ROH analysis a reduced WGS dataset of 23,875,519 SNPs with a minimum read depth of 3, a maximum read depth of 60 and a minimum mean read depth of 6 among 34 individuals (9 Mangalitza, three Mangalitza-crossbreeds and 22 individuals from nine pig breeds/populations; -minDP 3, -maxDP 60, -min-meanDP 6, -max-missing-count 30) was used. ROHs were defined as homozygous regions in sliding windows of a minimum of 20 SNPs within at least 2.4 kb with a density of 1 SNP per 0.12 kb (-homozyg-snp 20, -homozyg-density 0.12, –homozyg-kb 2.4). Within the sliding window, five SNPs with missing genotypes and three heterozygous SNPs as well as a distance between two homozygous SNPs not exceeding 100 kb were admitted (-homozyg-window-het 3, -homozyg-window-missing 5, -homozyg-gap 100). Detected ROHs were scanned for ROHR specifically found in all nine Mangalitza (for consensus ROH detection). In a next step, these ROHR were further narrowed down to those specific for Mangalitza by excluding ROHs present in the three Mangalitza-crossbreeds.

In addition to that, Porcine 50 K SNP chip genotyping data including 45,285 SNPs in 51 individuals (Mangalitza-crossbreeds, which were not F_1_-generation were excluded) was analyzed for ROHR specifically present in Mangalitza following similar steps like ROH analysis in WGS data. First, homozygous regions were defined as ROH by following conditions: a sliding window of a minimum of 20 SNPs with a density of 1 SNP per 1000 kb within at least 10 kb. Within sliding windows five SNPs with missing genotypes and three heterozygous SNPs as well as a homozygosity gap of a 100 kb were admitted for ROH detection. Identified ROHs were scanned for common ROHR specifically found in all Mangalitza (consensus ROH) or in at least eight of nineteen Mangalitza. Furthermore, detected regions were narrowed down by excluding those present in a minimum of five Mangalitza-crossbreeds.

ROH detection in WGS and SNP chip data was done using PLINK, version 1.09^79^ and requirements for ROH determination data were applied according to Bosse et al.^4^ and Herrero-Medrano et al.^5^. Detection of Mangalitza specific ROHR was performed using SAS/Genetics, version 9.4 (Statistical Analysis System, Cary, NC, USA).

Additionally, F_ROH_ were calculated for WGS data per individual as size of all ROH divided by the size of the autosomal genome captured with SNPs (2,265,395,079 bp). Breed specific F_ROH_ were indicated as mean of individual F_ROH_.

### Functional annotation of ROHR in Mangalitza

Functional annotation of genes in consensus ROHR exclusive for Mangalitza (SNP chip and WGS data) was carried out to detect biological processes and pathways potentially associated with breed-specific characteristics. An overlap of these ROHR with Ensemble genes was used to create a list of genes located in ROHR using SAS/Genetics, version 9.4 (Statistical Analysis System). PANTHER gene list analysis (version 15.0, http://pantherdb.org/)^80^ was applied to functionally assign genes in ROHR. Furthermore, we applied functional annotation for the detected genes based on Sscrofa11.1 background list using DAVID Bioinformatics Resources 6.8^81^. Fisher Exact test relying on the hypergeometrical distribution to compute P-values was performed. The EASE score threshold (maximum probability) for gene-enrichment analysis was set to 0.1. Gene enrichment analysis was based on 273 DAVID IDs (out of 668 genes) in WGS data and 184 (out of 483 genes) DAVID IDs for SNP chip genotyping data.

### Resource family

To investigate the segregation of curly hair phenotype in Mangalitza, mating of a curly-haired Mangalitza boar with a straight-haired Angeln Saddleback sow (MA × AS) as well as mating of a curly-haired Mangalitza sow with a straight-haired miniature pig boar (MA × MI) was performed. The Mangalitza boar and sow, used for the two matings, were derived from a litter of totally six pure-bred Mangalitza (2 female and 4 male). The development of curly hair was recorded in all progeny of both matings during their first year of life.

### Candidate SNPs and structural variants for curly hair

In order to identify potential candidate variants for the curly hair type in Mangalitza pigs, we used the total dataset of 69 pigs (see Supplementary Table S11). Those variants harboring one or two mutant alleles exclusively in a minimum of three Mangalitza and one Mangalitza-crossbreed but not in the straight-haired controls were extracted from the dataset using SAS/Genetics, version 9.4 (Statistical Analysis System). Then, the number of variants of interest was narrowed down by selecting only those variants with high or moderate effect predictions according to SNPEff. All identified variants were analyzed for their potential effects on proteins using the Sorting Intolerant From Tolerant (SIFT) pathogenicity prediction and score^82^ applied by the Ensemble Variant Effect Predictor Toolset^83^. Furthermore, PolyPhen-2^84^ predictions were performed for the two variants of interest.

Structural variants were identified using LUMPY software, version 0.2.13^85^, which integrated numerous structural variation signals within bam-files inferred from WGS data of 69 individuals (ten curly-haired Mangalitza, three Mangalitza-crossbreeds and 56 straight-haired controls). A generated VCF file including regions of 40 Mb distance to the two candidate SNPs for curly hair was screened for variants harboring one or two mutant alleles exclusively in Mangalitza and one mutant allele in Mangalitza-crossbreeds but not in the straight-haired controls using SAS (Statistical Analysis System).

### Validation of candidate SNPs and structural variants

Twelve candidate SNPs were validated in a first sample set of 85 individuals, including 23 curly-haired Mangalitza, 14 curly-haired Mangalitza-crossbreeds and 48 straight-haired individuals from twelve pig breeds/populations using restriction fragment length polymorphisms (RFLP) or competitive allele specific PCR (KASP) genotyping assays (LGC Genomics, Teddington, Middlesex, UK) according to the manufacturer’s protocol (see Supplementary Table S12). Allelic discrimination was conducted on an Applied Biosystems 7300 real-time PCR system (Applied Biosystems, Waltham, Massachusetts, USA). Six of these candidate SNPs, which were exclusively found in curly-haired Mangalitza, were further validated in 496 pigs, including 94 curly-haired Mangalitza and 48 curly-haired Mangalitza-crossbreeds and 354 straight-haired Mangalitza-crossbreeds or straight-haired individuals from different pig breeds/populations like 107 German Landrace, five German Landrace-crossbreeds, 108 German Large White, 18 Goettingen Minipig, 30 Leicoma, 64 European wild boar, 13 Bentheim Black Pied, two Husum Red Pied, four Mini-Lewe, one Pietrain, one Meishan and one Iberico.

In addition to that, the predicted tandem duplication on SCA13, visually examined using Integrative Genomics Viewer IGV (https://igv.org/), obtained by the search for structural variants exclusively in all Mangalitza and Mangalitza-crossbreeds but not in straight-haired controls was investigated using five TaqMan Copy Number assays (Thermo Fisher Scientific, Waltham, MA, USA) distributed across the predicted region to screen for copy number variation between curly and straight-haired individuals. We used *GCG* as housekeeping gene^86^ (Supplementary Table S13). Quantitative real-time PCR (qRT-PCR) was done in a reaction volume of totally 10.0 µl comprised of 2 ng genomic DNA, 5 µl TaqPath ProAmp Master Mix (Applied Biosystems, Foster City, CA, USA), 0.5 µl TaqMan Copy Number Assay (Applied Biosystems) and 2.5 µl water. The reaction was run for five curly-haired Mangalitza, two curly-haired Mangalitza-crossbreeds and three straight-haired individuals from three different pig breeds in triplex mode on ABI7300 under following conditions: an initial pre-read step at 60 °C for 30 s, one initial denaturation step at 95 °C for 5 min followed by 40 cycles at 95 °C for 5 s and annealing and extending at 60 °C for 30 s. The reaction was finalized with a post-read step at 60 °C for 30 s. Copy numbers were calculated using ΔΔCT method^87^ determining a copy number of two for straight-haired pigs as controls.

In addition, a gene fusion was validated in 189 curly-haired and 114 straight-haired individuals with PCR reactions producing an amplicon (predicted amplicon size 368 bp) spanning the fusion at the two expected breakpoints. A positive result represented the mutant allele whereas a negative result suggested the wild type allele (see Supplementary Fig. S1 and Supplementary Table S14).

### Validation of candidate SNPs in imputed data

For validation of the five candidate SNPs in a larger sample set, we used in addition to our genotyping data of 55 individuals run on the Genomic Profiler Porcine 50 K SNP chip (Illumina), further 2093 samples from pigs genotyped on 60 K SNP chip publicly available, representing 122 local and commercial breeds, 215 wild boars and 39 out-group suids^88^ (https://doi.org/10.5061/dryad.30tk6). Each breed was reviewed for its hair phenotype according to common literature. Breeds with unsecure curly phenotype were discarded, resulting in a total of 1336 individuals (61 Mangalitza/curly-haired Mangalitza-crossbreeds and 1275 straight-haired controls). This dataset was imputed using BEAGLE, version 5.1^89,90^ specifying effective population size at 1000. Imputed data set of 44,737,773 SNPs was narrowed down due to a required MAF of 0.05 using PLINK, version 1.09^79^. Subsequently, a total of 20,208,017 SNPs remained for screening genotype frequencies of imputed SNP alleles and genotypes applying SAS/Genetics, version 9.4 (Statistical Analysis System) procedures case–control and proc freq. An error rate for estimating the imputation accuracy was calculated as frequency of imputed genotypes differing from genotypes resulted from KASP genotyping.

## Supplementary Information

Supplementary Information 1.

Supplementary Table S1.

Supplementary Table S2.

Supplementary Table S7.

Supplementary Table S11.

## Data Availability

All necessary information needed to support the results can be found in the manuscript or are available from the corresponding author on reasonable request. Submission IDs for sequencing data are displayed in Supplementary Table S11.
