# Supplementary material for: Tracing selection signatures in the pig genome gives evidence for selective pressures on a unique curly hair phenotype in Mangalitza
Source: Sci Rep. 2020 Dec 17;10:22142. doi: 10.1038/s41598-020-79037-z (PMC7747725; doi:10.1038/s41598-020-79037-z)

**Tracing selection signatures in the pig genome gives evidence for selective pressures on a unique curly hair phenotype in Mangalitza**

Kathrin Schachler[1], Ottmar Distl[1], Julia Metzger[1,2*]

[1]Institute of Animal Breeding and Genetics, University of Veterinary Medicine Hannover, Hannover 30559, Germany

[2]Veterinary Functional Genomics Group, RG Development & Disease, Max Planck Institute for Molecular Genetics, Berlin 14195, Germany

*julia.metzger@tiho-hannover.de

## Supplementary Information

## Tables

**Table S1** Genes within consensus ROHR specific for Mangalitza breed inferred from WGS data. Chromosome (SCA), start and end of ROHR (bp), start and end of genes (bp) and gene IDs are presented.

**Table S2** Genes within consensus ROHR specific for Mangalitza breed inferred from SNP chip genotyping data. Chromosome (SCA), start and end of ROHR (bp), start and end of genes (bp) and gene IDs are presented.

**Table S3** Functional annotation for biological processes of genes within consensus ROHR specific for Mangalitza. PANTHER analysis was performed for ROHR inferred from WGS and SNP chip genotyping data. The proportion of gene hits against total genes and against the total number of process hits involved in distinct biological processes as well as the number of genes with gene names is presented.

| GO-Term | Analysis based on WGS | | | | Analysis based on SNP chip | | | |
|---|---|---|---|---|---|---|---|---|
| | Number of genes | Percent of gene hit against total #genes | Percent of gene hit against total #Process hits | Genes | Number of genes | Percent of gene hit against total #genes | Percent of gene hit against total # Process hits | Genes |
| behavior (GO:0007610) | 1 | 0.20% | 0.20% | *PLCB1* | - | - | - | - |
| biological adhesion (GO:0022610) | 14 | 3.00% | 2.20% | *PLA2G2D, IGSF21, PARD3, CDH10, PCDH15, BLK, CTNND2, CDH12, TENM2, SDK1, CDH5, PALLD, ENSSSCG00000009489, NTNG1* | 1 | 0.40% | 0.30% | CCN1 |
| biological regulation (GO:0065007) | 82 | 17.70% | 12.80% | *NFXL1, PLA2G2D, EHF, LDAH, ACKR2, COL4A2, LRCH1, LOC100523736, RAB31, ZFPM2, GPC5, ATP2C2, HECW1, KCNK2, MAST2, RBM20, PCGF5, GRM7, TIAM1, ARHGAP18, CCNT1, TBC1D5, NR1D2, HTR6, THRB, ADGRD1, ARHGAP15, UBP1, CHODL, RMND1, SLC4A7, PLCB1, DGKD, BLK, SUSD5, FBXL21P, MCTP1, LOC100512795, RETREG1, PLCG2, PLCL2, LAMTOR5, KCNK10, USP40, ATP8A2, GRIK1, PAXBP1, C10orf90, COTL1, SSTR2, DGKH, ZNF215, HOMER2, KLF3, LYPD6, NUB1, EVI5, RCAN1, CACNG5, XKR6, GPBP1L1, WFDC1, SRGAP3, GLRA3, HMBOX1, KSR2,* | 46 | 18.80% | 15.10% | *COL4A2, APC, CCNT2, ZP3, ING1, RAB3GAP1, CUX1, SNX3, KCNK2, VTCN1, ZC3H14, PHF10, HFM1, NR2E1, PKN2, ARHGAP15, DTX2, ATXN1L, BCL10, THBS2, GNAI2, ENSSSCG00000040692, SEMA3F, TBC1D16, TNRC6C, TENT4A, KCNK10, ENSSSCG00000040294, GRIK1, DDAH1, MAPKAPK3, ATP11A, BAIAP2, CALB2, SEMA3B, IRS2, CCN1, KLF6, ENSSSCG00000030031, BARHL2, SH2B2, RPTOR, ABRACL,* |

| | | | | | | | | |
|---|---|---|---|---|---|---|---|---|
| | | | | *RAB28, ENSSSCG00000009489, GRM1, CDC73, LOC100626731, CLSTN2, CNOT10, BANK1, CHN2, MAML3, FAM155A, MAPK10, CX3CR1, NRG2, CLEC16A, NKAIN2* | | | | *RAB20, ENSSSCG00000033862, FAM155A* |
| cell population proliferation (GO:0008283) | 2 | 0.40% | 0.30% | *PLA2G2D, BLK* | - | - | - | - |
| cellular component organization or biogenesis (GO:0071840) | 56 | 12.10% | 8.80% | *DNAJC6, DNAJC15, ENSSSCG00000035204, NEBL, UNC5C, PARD3, HECW1, CDH10, LOC100517188, MIS18A, COL14A1, MAST2, PCGF5, TIAM1, ARHGAP18, ENSSSCG00000005473, CHODL, SMYD3, ROBO2, LOXL2, BLK, SHQ1, ENSSSCG00000009595, FBXL21P, ENAH, RETREG1, CTNND2, CDH12, FRY, ATP8A2, SDK1, C10orf90, DYNC1LI1, COTL1, MAP7, ESPL1, EPB41L4A, TESK2, SMYD2, URB1, CAV3, CDH5, PALLD, SLIT2, CACNG5, SLIT3, XKR6, WFDC1, HMBOX1, ENSSSCG00000009489, CDC73, CLSTN2, TRAK1, GAS7, CLEC16A, NTNG1* | 22 | 9.00% | 7.20% | *APC, WDR37, CLVS1, ZNHIT6, SPAG17, WDR3, ROBO2, SEMA3F, TUBGCP3, ATP11A, SRP19, BAIAP2, FSCN2, WDR63, SEMA3B, ARFGEF3, RPTOR, TTC8, WDR36, TCHH, SEPT9, LIMK2* |
| cellular process (GO:0009987) | 159 | 34.30% | 24.90% | *KIN, NFXL1, PLA2G2D, EHF, DNAJC6, ACKR2, COL4A2, DNAJC15, LRCH1, ENSSSCG00000035204, RAB31, NEBL, ZFPM2, GPC5, UNC5C, CTSC, PARD3, ATP2C2, HECW1, CDH10, ENSSSCG00000038215, MIS18A, COL14A1, MAST2, PLA2G4A,* | 77 | 31.40% | 25.30% | *COL4A2, APC, CCNT2, ZP3, MAN1A2, ING1, ADARB2, CUX1, WDR37, NHSL1, CLVS1, CISH_tv2, VTCN1, ZC3H14, PHF10, CDC7, HFM1, NR2E1, ZNHIT6, PKN2, SPAG17, DTX2, PRDM8, CHST4,* |

*RBM20, ALPL, REV1, PCGF5, KIF15, GRM7, TIAM1, ARHGAP18, CCNT1, NR1D2, FDFT1, HTR6, FLT1, RPL31, THRB, ADGRD1, PLA2G5, FBXL2, AMPD1, UBP1, ENSSSCG00000005473, EXOSC7, MSRA, CHODL, MARCH3, CWH43, CHST4, MGST2, ENSSSCG00000031483, RMND1, SMYD3, SLC4A7, ROBO2, PTPRM, LOXL2, KIF13A, PLCB1, DGKD, BLK, SHQ1, SUSD5, ENSSSCG00000009595, FBXL21P, TUSC3, ENAH, MCTP1, ENSSSCG00000009182, RETREG1, UGT1A6, HBS1L, PLCG2, DPYSL5, NDST3, PLCL2, ART3, CTNND2, LAMTOR5, SIAH3, CDH12, USP40, TENM2, FRY, AARS, ATP8A2, GRIK1, EMC1, LSM2, SDK1, PAXBP1, C10orf90, DYNC1LI1, COTL1, SSTR2, MAP7, DGKH, ESPL1, ZNF215, ZDHHC7, EPB41L4A, TESK2, DNAH9, HOMER2, ZDHHC3, KLF3, LYPD6, DROSHA, SMYD2, MAGOH, URB1, NUB1, CAV3, CDH5, KIF26B, ELP3, PALLD, RCAN1, SLIT2, CACNG5, SLIT3, TRIM71, XKR6, GPBP1L1, XYLT1, WFDC1, SRGAP3, GLRA3, HMBOX1, ENSSSCG00000009503, KSR2, ALG14, RAB28, CARS2, ENSSSCG00000009489, GRM1, CDC73, ENSSSCG00000022462, CLSTN2, GXYLT2, FBXL7, PMS2, CNOT10, KIF13B,*

*ATXN1L, WDR3, MGAT5B, BCL10, ROBO2, GNAI2, ENSSSCG00000040692, SEMA3F, DRG1, SEC63, NSUN2, TUBGCP3, TNRC6C, SGSH, TENT4A, ENSSSCG00000040294, GK2, GRIK1, DDAH1, ENSSSCG00000004151, MAPKAPK3, NAA11, GBP2, ATP11A, SRP19, BAIAP2, GBP1, CALB2, NPLOC4, FSCN2, WDR63, ENSSSCG00000017146, SEMA3B, IRS2, ARFGEF3, CCN1, KLF6, BARHL2, SH2B2, RPTOR, CARS2, TTC8, WDR36, TCHH, ABRACL, KYNU, RAB20, ENSSSCG00000033862, SEPT9, FAM155A, MGAT5, LIMK2, GTF2B*

| | | | | | | | |
|---|---|---|---|---|---|---|---|
| | | | | *BANK1, TRAK1, MAML3, FAM155A, GAS7, OTUD3, MAPK10, HACL1, CX3CR1, NRG2, CLEC16A, NTNG1* | | | | |
| developmental process (GO:0032502) | 38 | 8.20% | 5.90% | *EHF, NEBL, ZFPM2, UNC5C, HECW1, CDH10, ENSSSCG00000038215, COL14A1, RBM20, TIAM1, NR1D2, FLT1, THRB, RBFOX1, CHODL, ROBO2, BLK, SGCZ, ENAH, CDH12, TENM2, FRY, ATP8A2, SDK1, ZNF215, CAV3, CDH5, PALLD, SLIT2, SLIT3, XKR6, ENSSSCG00000009489, CLSTN2, TRAK1, NAV2, GAS7, NRG2, NTNG1* | 15 | 6.10% | 4.90% | *RBFOX3, APC, ZP3, NHSL1, NR2E1, PRDM8, ATXN1L, THBS2, ROBO2, ENSSSCG00000040692, SGCZ, SEMA3F, SEMA3B, ENSSSCG00000030031, BARHL2* |
| growth (GO:0040007) | 3 | 0.60% | 0.50% | *ENSSSCG00000038215, COL14A1, WFDC1* | 3 | 1.20% | 1.00% | *SEMA3F, SEMA3B, RPTOR* |
| immune system process (GO:0002376) | 7 | 1.50% | 1.10% | *PLA2G2D, ACKR2, LRCH1, FLT1, BLK, BANK1, CX3CR1* | 5 | 2.00% | 1.60% | *VTCN1, BCL10, GBP2, GBP1, SH2B2,* |
| localization (GO:0051179) | 56 | 12.10% | 8.80% | *SLC39A11, DNAJC6, LDAH, ACKR2, DNAJC15, LRCH1, RAB31, GPC5, PARD3, SLC16A4, ATP2C2, HECW1, KCNK2, MIS18A, ENSSSCG00000028537, TBC1D5, ABCC12, PLA2G5, ENSSSCG00000005473, SLC4A7, PLCB1, BLK, ENAH, MCTP1, SLCO4C1, PLCG2, PLCL2, KCNK10, ATP8A2, ZDHHC7, HOMER2, ODR4, ZDHHC3, CAV3, EVI5, ANK1, ENSSSCG00000013057, CACNG5, ABCA4, SLC36A2, XKR6, SLC22A6, KPNA3,* | 24 | 9.80% | 7.90% | *APC, ANTXR2, CUX1, SNX3, KCNK2, SLC38A3, SLC38A10, ZNHIT6, SPAG17, SEMA3F, SEC63, TBC1D16, IST1, KCNK10, ATP11A, SRP19, FSCN2, WDR63, ORAI2, SEMA3B, TTC8, RAB20, SEPT9, FAM155A* |

| | | | | | | | | |
|---|---|---|---|---|---|---|---|---|
| | | | | *SRGAP3, GLRA3, RAB28, ENSSSCG00000009489, ENSSSCG00000029248, TRAK1, Kcns3, FAM155A, KCNG4, CX3CR1, CLEC16A, NTNG1, NKAIN2* | | | | |
| locomotion (GO:0040011) | 12 | 2.60% | 1.90% | *ACKR2, LRCH1, GPC5, UNC5C, ENAH, PLCG2, SLIT2, SLIT3, SRGAP3, ENSSSCG00000009489, CX3CR1, NTNG1* | 5 | 2.00% | 1.60% | *APC, SEMA3F, FSCN2, WDR63, SEMA3B* |
| metabolic process (GO:0008152) | 80 | 17.20% | 12.50% | *KIN, NFXL1, PLA2G2D, EHF, ZFPM2, LIPI, CTSC, HECW1, MAST2, PLA2G4A, RBM20, ALPL, REV1, PCGF5, CCNT1, NR1D2, FDFT1, RPL31, THRB, PLA2G5, FBXL2, AMPD1, UBP1, EXOSC7, MARCH3, CWH43, CHST4, MGST2, RMND1, SMYD3, PTPRM, LOXL2, DGKD, BLK, ENSSSCG00000009595, FBXL21P, TUSC3, ENSSSCG00000009182, RETREG1, UGT1A6, HBS1L, DPYSL5, NDST3, ART3, SIAH3, USP40, AARS, LSM2, PAXBP1, DGKH, ZNF215, ZDHHC7, HSD11B1, ZDHHC3, KLF3, DROSHA, SMYD2, MAGOH, URB1, NUB1, ELP3, CACNG5, TRIM71, GPBP1L1, XYLT1, HMBOX1, ENSSSCG00000009503, ALG14, CARS2, CDC73, ENSSSCG00000022462, GXYLT2, FBXL7, PMS2, CNOT10, MAML3, OTUD3, MAPK10, HACL1, CLEC16A* | 47 | 19.20% | 15.50% | *APC, CCNT2, MAN1A2, ING1, ADARB2, CUX1, WDR37, CISH_tv2, ZC3H14, PHF10, CDC7, HFM1, NR2E1, ZNHIT6, PKN2, DTX2, CHST4, ATXN1L, WDR3, MGAT5B, BCL10, ENSSSCG00000040692, DRG1, SEC63, NSUN2, TNRC6C, SGSH, TENT4A, ENSSSCG00000040294, GK2, DDAH1, ENSSSCG00000004151, MAPKAPK3, NAA11, SRD5A1, SRP19, ALKBH4, NPLOC4, ENSSSCG00000017146, KLF6, BARHL2, RPTOR, CARS2, WDR36, KYNU, MGAT5, GTF2B* |

| | | | | | | | | |
|---|---|---|---|---|---|---|---|---|
| multicellular organismal process (GO:0032501) | 41 | 8.80% | 6.40% | *NEBL, ZFPM2, UNC5C, HECW1, CDH10, ENSSSCG00000038215, COL14A1, RBM20, TAS1R2, TIAM1, NR1D2, FLT1, THRB, RBFOX1, CHODL, ROBO2, CLEC3B, PLCB1, BLK, SGCZ, ENAH, PLCG2, CDH12, TENM2, FRY, ATP8A2, SDK1, ZNF215, CDH5, PALLD, SLIT2, CACNG5, SLIT3, GLRA3, ENSSSCG00000009489, CLSTN2, TRAK1, NAV2, GAS7, NRG2, NTNG1* | 14 | 5.70% | 4.60% | *RBFOX3, APC, ZP3, VTCN1, PRDM8, THBS2, ROBO2, ENSSSCG00000040692, SGCZ, SEMA3F, SEMA3B, ENSSSCG00000030031, BARHL2, ENSSSCG00000033862* |
| multi-organism process (GO:0051704) | 1 | 0.20% | 0.20% | *LYG2,* | 3 | 1.20% | 1.00% | *ZP3, GBP2, GBP1* |
| reproduction (GO:0000003) | 1 | 0.20% | 0.20% | *ESPL1,* | 2 | 0.80% | 0.70% | *ZP3, TUBGCP3* |
| reproductive process (GO:0022414) | 1 | 0.20% | 0.20% | *ESPL1,* | 2 | 0.80% | 0.70% | *ZP3, TUBGCP3* |
| response to stimulus (GO:0050896) | 47 | 10.10% | 7.40% | *KIN, ACKR2, COL4A2, LRCH1, RAB31, GPC5, UNC5C, MAST2, REV1, GRM7, TIAM1, ARHGAP18, NR1D2, HTR6, THRB, ADGRD1, MSRA, PLCB1, DGKD, BLK, SUSD5, FBXL21P, ENAH, ENSSSCG00000009182, UGT1A6, PLCL2, LAMTOR5, SSTR2, DGKH, HOMER2, LYPD6, RCAN1, SLIT2, CACNG5, SLIT3, SLC36A2, LYG2, GLRA3, KSR2, RAB28, GRM1, PMS2, BANK1, MAML3, MAPK10, CX3CR1, NRG2* | 20 | 8.20% | 6.60% | *COL4A2, APC, VTCN1, CDC7, PKN2, DTX2, BCL10, GNAI2, SEMA3F, ENSSSCG00000004151, MAPKAPK3, GBP2, GBP1, SEMA3B, IRS2, CCN1, SH2B2, RPTOR, RAB20, ENSSSCG00000033862* |
| rhythmic process (GO:0048511) | 2 | 0.40% | 0.30% | *NR1D2, FBXL21P* | - | - | - | - |

| | | | | | | | | |
|---|---|---|---|---|---|---|---|---|
| signaling (GO:0023052) | 36 | 7.80% | 5.60% | *ACKR2*, *COL4A2*, *RAB31*, *GPC5*, *MAST2*, *GRM7*, *TIAM1*, *ARHGAP18*, *NR1D2*, *HTR6*, *THRB*, *ADGRD1*, *PLCB1*, *DGKD*, *BLK*, *SUSD5*, *MCTP1*, *PLCL2*, *LAMTOR5*, *GRIK1*, *SSTR2*, *DGKH*, *HOMER2*, *LYPD6*, *RCAN1*, *CACNG5*, *GLRA3*, *KSR2*, *RAB28*, *GRM1*, *CLSTN2*, *BANK1*, *MAML3*, *MAPK10*, *CX3CR1*, *NRG2* | 18 | 7.30% | 5.90% | *COL4A2*, *APC*, *VTCN1*, *PKN2*, *DTX2*, *BCL10*, *GNAI2*, *SEMA3F*, *GRIK1*, *MAPKAPK3*, *CALB2*, *SEMA3B*, *IRS2*, *CCN1*, *SH2B2*, *RPTOR*, *RAB20*, *ENSSSCG00000033862* |

**Table S4** Functional annotation for pathways of genes within consensus ROHR specific for Mangalitza. PANTHER analysis was performed for ROHR inferred from WGS and SNP chip genotyping data. The proportion of gene hits against total genes and against the total number of pathway hits that affect distinct pathways as well as the number of genes with gene names is presented.

| Pathway | Analysis based on WGS | | | | Analysis based on SNP chip | | | |
|---|---|---|---|---|---|---|---|---|
| | Number of genes | Percent of gene hit against total #genes | Percent of gene hit against total #Pathway hits | Genes | Number of genes | Percent of gene hit against total #genes | Percent of gene hit against total #Pathway hits | Genes |
| 2-arachidonoylglycerol biosynthesis (P05726) | 1 | 0.20% | 0.60% | *PLCB1* | 0- | - | - | - |
| 5HT1 type receptor mediated signaling pathway (P04373) | - | - | - | - | 1 | 0.40% | 1.50% | *GNAI2* |
| 5HT2 type receptor mediated signaling pathway (P04374) | 3 | 0.60% | 1.90% | *PLCB1, PLCG2, PLCL2* | - | - | - | - |
| 5-hydroxytryptamine degredation (P04372) | 1 | 0.20% | 0.60% | *ALDH5A1* | - | - | - | - |
| Alpha adrenergic receptor signaling pathway (P00002) | 1 | 0.20% | 0.60% | *PLCB1* | - | - | - | - |
| Alzheimer disease-amyloid secretase pathway (P00003) | 1 | 0.20% | 0.60% | *MAPK10* | 1 | 0.40% | 1.50% | *PKN2* |
| Aminobutyrate degradation (P02726) | 1 | 0.20% | 0.60% | *ALDH5A1* | - | - | - | - |
| Androgen/estrogene/progesterone biosynthesis (P02727) | 1 | 0.20% | 0.60% | *HSD17B2* | - | - | - | - |
| Angiogenesis (P00005) | 3 | 0.60% | 1.90% | *PLA2G4A, PLCG2, MAPK10* | 3 | 1.20% | 4.50% | *APC, MAPKAPK3, HSPB1* |
| Angiotensin II-stimulated signaling through G proteins and beta-arrestin (P05911) | 1 | 0.20% | 0.60% | *PLCB1* | - | - | - | - |

| | | | | | | | | |
|---|---|---|---|---|---|---|---|---|
| Apoptosis signaling pathway (P00006) | 1 | 0.20% | 0.60% | *MAPK10* | - | - | - | - |
| Axon guidance mediated by netrin (P00009) | 4 | 0.90% | 2.60% | *UNC5C, ENAH, PLCG2, NTNG1* | - | - | - | - |
| Axon guidance mediated by semaphorins (P00007) | 1 | 0.20% | 0.60% | *DPYSL5* | - | - | - | - |
| Axon guidance mediated by Slit/Robo (P00008) | 4 | 0.90% | 2.60% | *ENAH, SLIT2, SLIT3, NTNG1* | - | - | - | - |
| B cell activation (P00010) | 3 | 0.60% | 1.90% | *BLK, PLCG2, MAPK10* | - | - | - | - |
| Blood coagulation (P00011) | 1 | 0.20% | 0.60% | *FGG* | - | - | - | - |
| Cadherin signaling pathway (P00012) | 6 | 1.30% | 3.90% | *CDH10, CDH13, PCDH15, CTNND2, CDH12, CDH5* | - | - | - | - |
| CCKR signaling map (P06959) | 5 | 1.10% | 3.20% | *PLA2G4A, ARHGEF28, PLCB1, PRKG1, MAPK10* | 1 | 0.40% | 1.50% | *HSPB1* |
| Cholesterol biosynthesis (P00014) | 1 | 0.20% | 0.60% | *FDFT1* | 1 | 0.40% | 1.50% | *PDSS1* |
| Cytoskeletal regulation by Rho GTPase (P00016) | 3 | 0.60% | 1.90% | *ENSSSCG00000038144, ENAH, MYLK3* | 1 | 0.40% | 1.50% | *MYO3A* |
| Dopamine receptor mediated signaling pathway (P05912) | - | - | - | - | 1 | 0.40% | 1.50% | *GNAI2* |
| EGF receptor signaling pathway (P00018) | 5 | 1.10% | 3.20% | *MRPL38, MAP3K4, PLCG2, MAPK10, NRG2* | 2 | 0.80% | 3.00% | *YWHAG, RASA4B* |
| Endogenous cannabinoid signaling (P05730) | 2 | 0.40% | 1.30% | *PLCB1, GRM1* | - | - | - | - |
| Endothelin signaling pathway (P00019) | 3 | 0.60% | 1.90% | *PLA2G4A, PLCB1, PRKG1* | - | - | - | - |

| | | | | | | | | |
|---|---|---|---|---|---|---|---|---|
| Enkephalin release (P05913) | - | - | - | - | 1 | 0.40% | 1.50% | *GNAI2* |
| FAS signaling pathway (P00020) | 1 | 0.20% | 0.60% | *MAPK10* | - | - | - | - |
| FGF signaling pathway (P00021) | 3 | 0.60% | 1.90% | *MAP3K4, PLCG2, MAPK10* | 4 | 1.60% | 6.10% | *FGF5, FGF20, YWHAG, RASA4B* |
| Formyltetrahydroformate biosynthesis (P02743) | 1 | 0.20% | 0.60% | *MTHFD1L* | - | - | - | - |
| Gamma-aminobutyric acid synthesis (P04384) | 2 | 0.40% | 1.30% | *ALDH5A1, GAD2* | 1 | 0.40% | 1.50% | *GAD2* |
| General transcription regulation (P00023) | - | - | - | - | 1 | 0.40% | 1.50% | *GTF2B* |
| Gonadotropin-releasing hormone receptor pathway (P06664) | 4 | 0.90% | 2.60% | *PLA2G4A, MAP3K4, PLCB1, PCP4* | 2 | 0.80% | 3.00% | *GNAI2, IRS2* |
| Hedgehog signaling pathway (P00025) | - | - | - | - | 1 | 0.40% | 1.50% | *WDR37* |
| Heterotrimeric G-protein signaling pathway-Gi alpha and Gs alpha mediated pathway (P00026) | 5 | 1.10% | 3.20% | *GRM7, HTR6, SSTR2, RGS7, GRM1* | 1 | 0.40% | 1.50% | *GNAI2* |
| Heterotrimeric G-protein signaling pathway-Gq alpha and Go alpha mediated pathway (P00027) | 5 | 1.10% | 3.20% | *GRM7, PLCB1, SSTR2, RGS7, GRM1* | - | - | - | - |
| Heterotrimeric G-protein signaling pathway-rod outer segment phototransduction (P00028) | 1 | 0.20% | 0.60% | *PDC* | - | - | - | - |
| Histamine H1 receptor mediated signaling pathway (P04385) | 3 | 0.60% | 1.90% | *PLCB1, PLCG2, PLCL2* | - | - | - | - |
| Histidine biosynthesis (P02747) | 1 | 0.20% | 0.60% | *TAT* | 1 | 0.40% | 1.50% | *TAT* |
| Huntington disease (P00029) | 4 | 0.90% | 2.60% | *CAPN7, GRIK1, DYNC1LI1, VAT1L* | 1 | 0.40% | 1.50% | *GRIK1* |
| Hypoxia response via HIF activation (P00030) | - | - | - | - | 1 | 0.40% | 1.50% | *RORC* |

| | | | | | | | | |
|---|---|---|---|---|---|---|---|---|
| Inflammation mediated by chemokine and cytokine signaling pathway (P00031) | 9 | 1.90% | 5.80% | *COL14A1*, *PLA2G4A*, *ENSSSCG00000038144*, *MAP3K4*, *PLCB1*, *PLCG2*, *PLCL2*, *MYLK3*, *CX3CR1* | 3 | 1.20% | 4.50% | *CISH_tv2*, *GNAI2*, *MYO3A* |
| Insulin/IGF pathway-mitogen activated protein kinase kinase/MAP kinase cascade (P00032) | 1 | 0.20% | 0.60% | *RPS6KA4* | 1 | 0.40% | 1.50% | *IRS2* |
| Insulin/IGF pathway-protein kinase B signaling cascade (P00033) | - | - | - | - | 1 | 0.40% | 1.50% | *IRS2* |
| Integrin signalling pathway (P00034) | 8 | 1.70% | 5.20% | *COL4A2*, *COL14A1*, *MAP3K4*, *DOCK1*, *ITGA8*, *COL4A3*, *COL4A4*, *MAPK10* | 1 | 0.40% | 1.50% | *COL4A2* |
| Interferon-gamma signaling pathway (P00035) | 1 | 0.20% | 0.60% | *MAPK10* | 1 | 0.40% | 1.50% | *CISH_tv2* |
| Interleukin signaling pathway (P00036) | 1 | 0.20% | 0.60% | *MAP3K4* | 1 | 0.40% | 1.50% | *IRS2* |
| Ionotropic glutamate receptor pathway (P00037) | 2 | 0.40% | 1.30% | *GRIK1*, *CACNG5* | 1 | 0.40% | 1.50% | *GRIK1* |
| Metabotropic glutamate receptor group I pathway (P00041) | 2 | 0.40% | 1.30% | *GRIK1*, *GRM1* | 1 | 0.40% | 1.50% | *GRIK1* |
| Metabotropic glutamate receptor group II pathway (P00040) | - | - | - | - | 1 | 0.40% | 1.50% | *GNAI2* |
| Metabotropic glutamate receptor group III pathway (P00039) | 3 | 0.60% | 1.90% | *GRM7*, *GRIK1*, *GRM1* | 2 | 0.80% | 3.00% | *GNAI2*, *GRIK1* |
| Methylmalonyl pathway (P02755) | 1 | 0.20% | 0.60% | *PCCA* | - | - | - | - |
| Muscarinic acetylcholine receptor 1 and 3 signaling pathway (P00042) | - | - | - | - | 1 | 0.40% | 1.50% | *PKN2* |

| | | | | | | | | |
|---|---|---|---|---|---|---|---|---|
| Muscarinic acetylcholine receptor 2 and 4 signaling pathway (P00043) | - | - | - | - | 1 | 0.40% | 1.50% | *GNAI2* |
| Nicotine degradation (P05914) | 1 | 0.20% | 0.60% | *UGT1A6* | - | - | - | - |
| Nicotinic acetylcholine receptor signaling pathway (P00044) | 3 | 0.60% | 1.90% | *ENSSSCG00000038144, MYO10, SLC44A3* | 1 | 0.40% | 1.50% | *MYO3A* |
| Opioid prodynorphin pathway (P05916) | - | - | - | - | 1 | 0.40% | 1.50% | *GNAI2* |
| Opioid proenkephalin pathway (P05915) | - | - | - | - | 1 | 0.40% | 1.50% | *GNAI2* |
| Opioid proopiomelanocortin pathway (P05917) | - | - | - | - | 1 | 0.40% | 1.50% | *GNAI2* |
| Oxidative stress response (P00046) | 3 | 0.60% | 1.90% | *PLA2G4A, MAP3K4, MAPK10* | - | - | - | - |
| Oxytocin receptor mediated signaling pathway (P04391) | 3 | 0.60% | 1.90% | *PLCB1, PLCG2, PLCL2* | - | - | - | - |
| p38 MAPK pathway (P05918) | 2 | 0.40% | 1.30% | *RPS6KA4, MAP3K4* | 2 | 0.80% | 3.00% | *MAPKAPK3, HSPB1* |
| Parkinson disease (P00049) | 2 | 0.40% | 1.30% | *BLK, MAPK10* | 1 | 0.40% | 1.50% | *YWHAG* |
| PDGF signaling pathway (P00047) | 8 | 1.70% | 5.20% | *EHF, RPS6KA4, ARHGAP15, MAP3K4, STARD13, PLCG2, SRGAP3, MAPK10* | 3 | 1.20% | 4.50% | *PKN2, ARHGAP15, RASA4B* |
| PI3 kinase pathway (P00048) | - | - | - | - | 1 | 0.40% | 1.50% | *GNAI2* |
| Plasminogen activating cascade (P00050) | 1 | 0.20% | 0.60% | *FGG* | - | - | - | - |
| Pyrimidine metabolism (P02771) | 2 | 0.40% | 1.30% | *DPYD, DPYSL5* | - | - | - | - |
| Ras pathway (P04393) | 3 | 0.60% | 1.90% | *TIAM1, MAP3K4, MAPK10* | 2 | 0.80% | 3.00% | *MAP3K7CL, MAPKAPK3* |
| T cell activation (P00053) | 1 | 0.20% | 0.60% | *MAPK10* | - | - | - | - |

| | | | | | | | | |
|---|---|---|---|---|---|---|---|---|
| TGF-beta signaling pathway (P00052) | 1 | 0.20% | 0.60% | *MAPK10* | 2 | 0.80% | 3.00% | *MAP3K7CL*, *CITED2* |
| Thyrotropin-releasing hormone receptor signaling pathway (P04394) | 3 | 0.60% | 1.90% | *PLCB1*, *PLCG2*, *PLCL2* | - | - | - | - |
| Toll receptor signaling pathway (P00054) | 1 | 0.20% | 0.60% | *MAPK10* | 1 | 0.40% | 1.50% | *ENSSSCG00000004151* |
| Tyrosine biosynthesis (P02784) | 1 | 0.20% | 0.60% | *TAT* | 1 | 0.40% | 1.50% | *TAT* |
| Transcription regulation by bZIP transcription factor (P00055) | - | - | - | - | 2 | 0.80% | 3.00% | *TTF2*, *GTF2B* |
| Ubiquitin proteasome pathway (P00060) | - | - | - | - | 1 | 0.40% | 1.50% | *ENSSSCG00000004151* |
| VEGF signaling pathway (P00056) | 2 | 0.40% | 1.30% | *PLA2G4A*, *PLCG2* | 2 | 0.80% | 3.00% | *MAPKAPK3*, *HSPB1* |
| Wnt signaling pathway (P00057) | 7 | 1.50% | 4.50% | *CDH10*, *CDH13*, *PCDH15*, *CTBP2*, *PLCB1*, *CDH12*, *CDH5* | 4 | 1.60% | 6.10% | *APC*, *MAP3K7CL*, *WDR37*, *CHD1L* |

**Table S5** Functional annotation performed with DAVID Bioinformatics Resources. DAVID analysis was executed for ROHR inferred from WGS data and SNP chip genotyping data. EASE score threshold for gene enrichment-analysis was set to 0.1. In total, 273 DAVID ID records are included for WGS data and 184 for SNP chip genotyping data.

| Category | Term | Analysis based on WGS | | | | Analysis based on SNP chip | | | |
|---|---|---|---|---|---|---|---|---|---|
| | | Count | % | P-Value | Fisher Exact | Count | % | P-Value | Fisher Exact |
| GOTERM_BP_DIRECT | 2-oxoglutarate metabolic process | - | - | - | - | 2 | 1.1 | 9.50E-02 | 4.20E-03 |
| INTERPRO | Aldolase-type TIM barrel | - | - | - | - | 3 | 1.6 | 6.10E-02 | 7.70E-03 |
| GOTERM_CC_DIRECT | anchored component of plasma membrane | - | - | - | - | 2 | 1.1 | 8.70E-02 | 3.50E-03 |
| INTERPRO | Anoctamin/TMEM 16 | 4 | 1.5 | 7.30E-05 | 8.70E-07 | - | - | - | - |
| INTERPRO | Appr-1-p processing | - | - | - | - | 2 | 1.1 | 9.20E-02 | 3.90E-03 |
| KEGG_PATHWAY | Axon guidance | 5 | 1.8 | 5.80E-02 | 1.60E-02 | 5 | 2.7 | 3.70E-02 | 8.70E-03 |
| GOTERM_CC_DIRECT | axoneme | - | - | - | - | 3 | 1.6 | 5.10E-02 | 5.70E-03 |
| SMART | C1 | 4 | 1.5 | 3.80E-02 | 6.50E-03 | - | - | - | - |
| INTERPRO | C2 calcium-dependent membrane targeting | - | - | - | - | 5 | 2.7 | 2.40E-02 | 4.90E-03 |
| SMART | CA | 4 | 1.5 | 7.10E-02 | 1.60E-02 | - | - | - | - |
| INTERPRO | Cadherin | 4 | 1.5 | 6.20E-02 | 1.30E-02 | - | - | - | - |
| INTERPRO | Cadherin-like | 4 | 1.5 | 6.50E-02 | 1.40E-02 | - | - | - | - |
| GOTERM_BP_DIRECT | calcium activated galactosylceramide scrambling | 2 | 0.7 | 2.80E-02 | 2.00E-04 | - | - | - | - |
| GOTERM_BP_DIRECT | calcium activated phosphatidylcholine scrambling | 2 | 0.7 | 2.80E-02 | 2.00E-04 | - | - | - | - |
| GOTERM_MF_DIRECT | calcium ion binding | 12 | 4.4 | 8.40E-02 | 4.50E-02 | - | - | - | - |
| INTERPRO | Calcium-activated chloride channel protein | 2 | 0.7 | 4.60E-02 | 7.30E-04 | 3 | 1.6 | 3.30E-04 | 1.20E-06 |
| GOTERM_BP_DIRECT | carbohydrate metabolic process | - | - | - | - | 5 | 2.7 | 3.00E-02 | 6.60E-03 |
| GOTERM_BP_DIRECT | cartilage development | - | - | - | - | 3 | 1.6 | 2.40E-02 | 1.80E-03 |
| GOTERM_CC_DIRECT | cell junction | 6 | 2.2 | 1.50E-02 | 3.20E-03 | 4 | 2.2 | 7.40E-02 | 1.70E-02 |
| GOTERM_BP_DIRECT | cellular amino acid metabolic process | - | - | - | - | 2 | 1.1 | 9.50E-02 | 4.20E-03 |
| GOTERM_BP_DIRECT | cellular response to interleukin-1 | - | - | - | - | 3 | 1.6 | 4.60E-02 | 4.90E-03 |
| GOTERM_BP_DIRECT | cellular response to platelet-derived growth factor stimulus | - | - | - | - | 2 | 1.1 | 9.50E-02 | 4.20E-03 |

| | | | | | | | | | |
|---|---|---|---|---|---|---|---|---|---|
| GOTERM_BP_DIRECT | cellular response to transforming growth factor beta stimulus | 3 | 1.1 | 2.70E-02 | 2.00E-03 | - | - | - | - |
| GOTERM_BP_DIRECT | cellular response to tumor necrosis factor | - | - | - | - | 3 | 1.6 | 7.60E-02 | 1.10E-02 |
| GOTERM_BP_DIRECT | cellular response to UV-B | - | - | - | - | 3 | 1.6 | 1.20E-03 | 1.30E-05 |
| INTERPRO | Chloride channel calcium-activated | 2 | 0.7 | 4.60E-02 | 7.30E-04 | 3 | 1.6 | 3.30E-04 | 1.20E-06 |
| GOTERM_BP_DIRECT | chloride transmembrane transport | - | - | - | - | 3 | 1.6 | 4.10E-02 | 4.00E-03 |
| KEGG_PATHWAY | Choline metabolism in cancer | 6 | 2.2 | 6.60E-03 | 1.20E-03 | - | - | - | - |
| INTERPRO | Choline transporter-like | 2 | 0.7 | 7.60E-02 | 2.40E-03 | - | - | - | - |
| INTERPRO | Chromo domain/shadow | - | - | - | - | 3 | 1.6 | 2.70E-02 | 2.10E-03 |
| INTERPRO | Chromo domain-like | - | - | - | - | 3 | 1.6 | 2.30E-02 | 1.60E-03 |
| GOTERM_CC_DIRECT | clathrin-coated vesicle | - | - | - | - | 3 | 1.6 | 1.70E-02 | 1.00E-03 |
| UP_KEYWORDS | Coiled coil | 37 | 13.6 | 7.50E-02 | 5.50E-02 | 30 | 16.3 | 1.80E-02 | 1.10E-02 |
| INTERPRO | CRAL/TRIO, N-terminal domain | - | - | - | - | 2 | 1.1 | 9.20E-02 | 3.90E-03 |
| INTERPRO | CRAL-TRIO domain | - | - | - | - | 3 | 1.6 | 2.30E-02 | 1.60E-03 |
| GOTERM_CC_DIRECT | cytoplasm | 41 | 15 | 6.80E-02 | 5.00E-02 | - | - | - | - |
| GOTERM_CC_DIRECT | cytoplasmic vesicle | - | - | - | - | 4 | 2.2 | 2.90E-02 | 4.50E-03 |
| GOTERM_BP_DIRECT | cytoskeleton-dependent intracellular transport | 3 | 1.1 | 8.40E-03 | 3.20E-04 | - | - | - | - |
| UP_KEYWORDS | Decarboxylase | - | - | - | - | 2 | 1.1 | 7.10E-02 | 2.20E-03 |
| UP_KEYWORDS | Detoxification | 2 | 0.7 | 4.50E-02 | 6.90E-04 | - | - | - | - |
| SMART | DEXDc | - | - | - | - | 5 | 2.7 | 2.20E-02 | 4.40E-03 |
| UP_SEQ_FEATURE | domain:EGF-like | - | - | - | - | 2 | 1.1 | 6.20E-02 | 1.90E-03 |
| UP_SEQ_FEATURE | domain:VWFA | 2 | 0.7 | 8.30E-02 | 3.30E-03 | - | - | - | - |
| INTERPRO | EF-Hand 1, calcium-binding site | - | - | - | - | 6 | 3.3 | 2.00E-02 | 5.00E-03 |
| INTERPRO | EF-hand domain | - | - | - | - | 6 | 3.3 | 4.40E-02 | 1.30E-02 |
| INTERPRO | EF-hand-like domain | - | - | - | - | 7 | 3.8 | 3.50E-02 | 1.10E-02 |
| INTERPRO | Extracellular ligand-binding receptor | 3 | 1.1 | 6.60E-02 | 8.50E-03 | - | - | - | - |
| GOTERM_CC_DIRECT | fibrinogen complex | 3 | 1.1 | 4.40E-03 | 1.10E-04 | - | - | - | - |
| INTERPRO | Fibrinogen, alpha/beta/gamma chain, coiled coil domain | 3 | 1.1 | 1.40E-03 | 1.50E-05 | - | - | - | - |
| INTERPRO | Forkhead-associated (FHA) domain | 4 | 1.5 | 8.40E-03 | 7.90E-04 | - | - | - | - |
| KEGG_PATHWAY | Gastric acid secretion | 4 | 1.5 | 5.80E-02 | 1.20E-02 | - | - | - | - |
| INTERPRO | Glucose/ribitol dehydrogenase | 4 | 1.5 | 4.30E-02 | 7.80E-03 | - | - | - | - |
| KEGG_PATHWAY | Glutamatergic synapse | 6 | 2.2 | 1.70E-02 | 4.00E-03 | - | - | - | - |
| KEGG_PATHWAY | Glycosaminoglycan biosynthesis - heparan sulfate / heparin | 3 | 1.1 | 3.50E-02 | 3.10E-03 | - | - | - | - |
| KEGG_PATHWAY | Glycosaminoglycan degradation | - | - | - | - | 4 | 2.2 | 9.70E-04 | 4.20E-05 |

| | | | | | | | | | |
|---|---|---|---|---|---|---|---|---|---|
| UP_KEYWORDS | Glycosidase | - | - | - | - | 5 | 2.7 | 1.00E-03 | 8.40E-05 |
| INTERPRO | Glycoside hydrolase, superfamily | - | - | - | - | 4 | 2.2 | 1.40E-02 | 1.60E-03 |
| INTERPRO | GPCR, family 3, metabotropic glutamate receptor | 2 | 0.7 | 9.00E-02 | 3.50E-03 | - | - | - | - |
| INTERPRO | Helicase, C-terminal | - | - | - | - | 5 | 2.7 | 1.50E-02 | 2.60E-03 |
| INTERPRO | Helicase, superfamily 1/2, ATP-binding domain | - | - | - | - | 5 | 2.7 | 1.50E-02 | 2.60E-03 |
| SMART | HELICc | - | - | - | - | 5 | 2.7 | 2.40E-02 | 4.90E-03 |
| GOTERM_BP_DIRECT | hematopoietic progenitor cell differentiation | - | - | - | - | 3 | 1.6 | 8.50E-02 | 1.30E-02 |
| GOTERM_BP_DIRECT | homophilic cell adhesion via plasma membrane adhesion molecules | 4 | 1.5 | 8.30E-02 | 2.00E-02 | - | - | - | - |
| GOTERM_BP_DIRECT | hyaluronan catabolic process | - | - | - | - | 3 | 1.6 | 7.20E-04 | 5.40E-06 |
| PIR_SUPERFAMILY | hyaluronidase | - | - | - | - | 3 | 1.6 | 1.20E-03 | 1.50E-05 |
| INTERPRO | Hyaluronidase | - | - | - | - | 3 | 1.6 | 1.10E-03 | 1.20E-05 |
| GOTERM_MF_DIRECT | hyaluronoglucuronidase activity | - | - | - | - | 2 | 1.1 | 2.00E-02 | 1.00E-04 |
| GOTERM_MF_DIRECT | hyalurononglucosaminidase activity | - | - | - | - | 3 | 1.6 | 1.50E-03 | 2.00E-05 |
| INTERPRO | Immunoglobulin V-set | - | - | - | - | 5 | 2.7 | 3.50E-02 | 8.10E-03 |
| INTERPRO | Immunoglobulin-like domain | - | - | - | - | 8 | 4.3 | 8.90E-02 | 4.00E-02 |
| GOTERM_CC_DIRECT | immunological synapse | 3 | 1.1 | 4.90E-02 | 5.20E-03 | - | - | - | - |
| GOTERM_MF_DIRECT | inorganic anion exchanger activity | 5 | 1.8 | 8.90E-05 | 3.40E-06 | - | - | - | - |
| GOTERM_BP_DIRECT | inorganic anion transport | 2 | 0.7 | 4.20E-02 | 6.10E-04 | - | - | - | - |
| KEGG_PATHWAY | Inositol phosphate metabolism | 4 | 1.5 | 4.90E-02 | 9.40E-03 | - | - | - | - |
| GOTERM_CC_DIRECT | integral component of plasma membrane | 21 | 7.7 | 3.10E-03 | 1.40E-03 | - | - | - | - |
| INTERPRO | Intermediate filament head, DNA-binding domain | 2 | 0.7 | 9.00E-02 | 3.50E-03 | - | - | - | - |
| GOTERM_MF_DIRECT | intracellular calcium activated chloride channel activity | 5 | 1.8 | 5.20E-06 | 7.70E-08 | 3 | 1.6 | 3.50E-03 | 8.40E-05 |
| GOTERM_BP_DIRECT | intracellular signal transduction | 7 | 2.6 | 7.60E-02 | 3.00E-02 | - | - | - | - |
| UP_KEYWORDS | Ion transport | 9 | 3.3 | 3.90E-02 | 1.60E-02 | - | - | - | - |
| INTERPRO | Kinesin protein 1B | 2 | 0.7 | 6.10E-02 | 1.40E-03 | - | - | - | - |
| INTERPRO | Kinesin, motor domain | 3 | 1.1 | 9.90E-02 | 1.60E-02 | - | - | - | - |
| INTERPRO | Kinesin-like | 2 | 0.7 | 7.60E-02 | 2.40E-03 | - | - | - | - |
| INTERPRO | Major facilitator superfamily domain | 6 | 2.2 | 4.80E-02 | 1.50E-02 | - | - | - | - |
| KEGG_PATHWAY | Metabolic pathways | 24 | 8.8 | 1.10E-02 | 6.40E-03 | - | - | - | - |
| UP_KEYWORDS | Metal-binding | - | - | - | - | 18 | 9.8 | 7.20E-02 | 4.40E-02 |
| GOTERM_MF_DIRECT | microtubule motor activity | 4 | 1.5 | 2.40E-02 | 3.50E-03 | - | - | - | - |

| | | | | | | | | | |
|---|---|---|---|---|---|---|---|---|---|
| GOTERM_BP_DIRECT | microtubule-based movement | 4 | 1.5 | 2.60E-02 | 3.80E-03 | - | - | - | - |
| GOTERM_CC_DIRECT | microvillus | - | - | - | - | 3 | 1.6 | 3.60E-02 | 3.40E-03 |
| GOTERM_BP_DIRECT | multicellular organism aging | - | - | - | - | 2 | 1.1 | 9.50E-02 | 4.20E-03 |
| UP_KEYWORDS | NADP | 5 | 1.8 | 1.30E-02 | 2.20E-03 | - | - | - | - |
| GOTERM_CC_DIRECT | neurofilament | 2 | 0.7 | 8.60E-02 | 3.20E-03 | - | - | - | - |
| GOTERM_MF_DIRECT | neuropilin binding | - | - | - | - | 2 | 1.1 | 6.90E-02 | 2.10E-03 |
| GOTERM_BP_DIRECT | olfactory bulb development | - | - | - | - | 3 | 1.6 | 5.20E-03 | 1.50E-04 |
| KEGG_PATHWAY | Phosphatidylinositol signaling system | 5 | 1.8 | 3.00E-02 | 6.70E-03 | - | - | - | - |
| GOTERM_MF_DIRECT | phospholipid scramblase activity | 2 | 0.7 | 8.40E-02 | 3.10E-03 | - | - | - | - |
| GOTERM_CC_DIRECT | photoreceptor outer segment | 3 | 1.1 | 7.20E-02 | 9.80E-03 | - | - | - | - |
| GOTERM_BP_DIRECT | platelet activation | 3 | 1.1 | 3.20E-02 | 2.80E-03 | - | - | - | - |
| KEGG_PATHWAY | Platelet activation | 6 | 2.2 | 2.10E-02 | 5.40E-03 | - | - | - | - |
| INTERPRO | Pleckstrin homology-like domain | 9 | 3.3 | 8.50E-02 | 4.00E-02 | - | - | - | - |
| INTERPRO | P-loop containing nucleoside triphosphate hydrolase | - | - | - | - | 14 | 7.6 | 3.20E-02 | 1.60E-02 |
| GOTERM_MF_DIRECT | poly(A) binding | - | - | - | - | 2 | 1.1 | 8.80E-02 | 3.60E-03 |
| GOTERM_BP_DIRECT | positive regulation of cell-cell adhesion | 2 | 0.7 | 9.60E-02 | 4.10E-03 | - | - | - | - |
| GOTERM_BP_DIRECT | positive regulation of interleukin-8 secretion | - | - | - | - | 2 | 1.1 | 8.50E-02 | 3.30E-03 |
| GOTERM_BP_DIRECT | positive regulation of translation | - | - | - | - | 3 | 1.6 | 3.80E-02 | 3.60E-03 |
| GOTERM_CC_DIRECT | PRC1 complex | - | - | - | - | 2 | 1.1 | 9.60E-02 | 4.30E-03 |
| INTERPRO | Protein kinase C-like, phorbol ester/diacylglycerol binding | 4 | 1.5 | 3.30E-02 | 5.40E-03 | - | - | - | - |
| GOTERM_BP_DIRECT | protein polymerization | 3 | 1.1 | 5.30E-03 | 1.50E-04 | - | - | - | - |
| GOTERM_MF_DIRECT | pyridoxal phosphate binding | - | - | - | - | 4 | 2.2 | 9.90E-03 | 1.00E-03 |
| INTERPRO | Pyridoxal phosphate-dependent transferase | - | - | - | - | 4 | 2.2 | 7.00E-03 | 6.30E-04 |
| INTERPRO | Pyridoxal phosphate-dependent transferase, major region, subdomain 1 | - | - | - | - | 4 | 2.2 | 7.00E-03 | 6.30E-04 |
| INTERPRO | Pyridoxal phosphate-dependent transferase, major region, subdomain 2 | - | - | - | - | 4 | 2.2 | 5.10E-03 | 4.00E-04 |
| KEGG_PATHWAY | Renin secretion | - | - | - | - | 4 | 2.2 | 3.40E-02 | 5.60E-03 |
| GOTERM_BP_DIRECT | response to amino acid | 2 | 0.7 | 9.60E-02 | 4.10E-03 | - | - | - | - |
| GOTERM_BP_DIRECT | response to antibiotic | - | - | - | - | 3 | 1.6 | 1.20E-02 | 5.60E-04 |
| GOTERM_BP_DIRECT | response to reactive oxygen species | - | - | - | - | 2 | 1.1 | 6.50E-02 | 1.80E-03 |
| GOTERM_BP_DIRECT | response to virus | - | - | - | - | 3 | 1.6 | 6.30E-02 | 8.00E-03 |
| GOTERM_BP_DIRECT | Roundabout signaling pathway | 2 | 0.7 | 8.30E-02 | 3.00E-03 | - | - | - | - |

| | | | | | | | | | |
|---|---|---|---|---|---|---|---|---|---|
| INTERPRO | S100/CaBP-9k-type, calcium binding, subdomain | - | - | - | - | 5 | 2.7 | 1.10E-04 | 4.70E-06 |
| INTERPRO | S100/Calbindin-D9k, conserved site | - | - | - | - | 3 | 1.6 | 1.50E-02 | 8.70E-04 |
| SMART | SEC14 | - | - | - | - | 3 | 1.6 | 2.30E-02 | 1.60E-03 |
| INTERPRO | Sec63 domain | - | - | - | - | 2 | 1.1 | 2.10E-02 | 1.10E-04 |
| GOTERM_BP_DIRECT | self proteolysis | 2 | 0.7 | 6.90E-02 | 2.00E-03 | - | - | - | - |
| GOTERM_MF_DIRECT | signal transducer activity | - | - | - | - | 4 | 2.2 | 7.40E-02 | 1.70E-02 |
| GOTERM_BP_DIRECT | signal transduction | 11 | 4 | 2.80E-02 | 1.20E-02 | - | - | - | - |
| SMART | SM00973 | - | - | - | - | 2 | 1.1 | 2.50E-02 | 1.60E-04 |
| SMART | SM01100 | - | - | - | - | 2 | 1.1 | 9.50E-02 | 4.20E-03 |
| SMART | SM01212 | 3 | 1.1 | 1.60E-03 | 1.80E-05 | - | - | - | - |
| SMART | SM01394 | - | - | - | - | 5 | 2.7 | 2.00E-04 | 9.90E-06 |
| INTERPRO | SMAD/FHA domain | 5 | 1.8 | 4.10E-03 | 4.90E-04 | - | - | - | - |
| INTERPRO | SNF2-related | - | - | - | - | 3 | 1.6 | 2.90E-02 | 2.30E-03 |
| GOTERM_MF_DIRECT | sodium-independent organic anion transmembrane transporter activity | 5 | 1.8 | 6.90E-05 | 2.40E-06 | - | - | - | - |
| GOTERM_BP_DIRECT | sodium-independent organic anion transport | 5 | 1.8 | 8.20E-05 | 3.10E-06 | - | - | - | - |
| INTERPRO | Sterile alpha motif/pointed domain | 5 | 1.8 | 3.40E-02 | 7.70E-03 | - | - | - | - |
| INTERPRO | Thrombospondin, type 1 repeat | - | - | - | - | 3 | 1.6 | 8.60E-02 | 1.30E-02 |
| KEGG_PATHWAY | Tryptophan metabolism | - | - | - | - | 3 | 1.6 | 6.60E-02 | 8.80E-03 |
| INTERPRO | Two pore domain potassium channel, TREK | 2 | 0.7 | 3.10E-02 | 2.50E-04 | 2 | 1.1 | 2.10E-02 | 1.10E-04 |
| INTERPRO | von Willebrand factor, type A | 5 | 1.8 | 2.10E-02 | 4.00E-03 | 5 | 2.7 | 5.60E-03 | 7.40E-04 |
| SMART | VWA | 4 | 1.5 | 4.20E-02 | 7.60E-03 | 5 | 2.7 | 2.70E-03 | 2.90E-04 |
| INTERPRO | WD40 repeat | - | - | - | - | 6 | 3.3 | 7.00E-02 | 2.40E-02 |
| INTERPRO | WD40 repeat, conserved site | - | - | - | - | 5 | 2.7 | 4.90E-02 | 1.20E-02 |
| INTERPRO | WD40/YVTN repeat-like-containing domain | - | - | - | - | 8 | 4.3 | 1.70E-02 | 5.30E-03 |
| INTERPRO | WD40-repeat-containing domain | - | - | - | - | 6 | 3.3 | 1.00E-01 | 3.80E-02 |
| GOTERM_MF_DIRECT | zinc ion binding | - | - | - | - | 14 | 7.6 | 5.80E-02 | 3.10E-02 |

**Table S6** Phenotype of hair coat in Mangalitza crossbreeds. Number of samples of the particular crossing (n) as well as the amount of straight and curly -haired individuals is indicated. Square symbols represent male individuals, circles female and rhombus individuals of unknown sex. A curly hair phenotype is displayed by filled symbols, whereas unfilled symbols signify straight hair.

| **Crossing generation** | | **Population** | **n** | **straight-haired (n)** | **curly-haired (n)** |
|---|---|---|---|---|---|
| F1 | 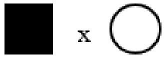 | Mangalitza x Angeln Saddleback | 12 | 0 | 12 |
| | | Mangalitza x Bentheim Black Pied | 19 | 0 | 19 |
| | 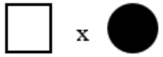 | Mangalitza x Miniature pig | 9 | 0 | 9 |
| | | Mangalitza x European Wild Boar | 6 | 0 | 6 |
| F2 | 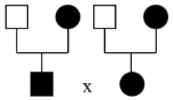 | [Mangalitza x European Wild Boar] x [Mangalitza x European Wild Boar] | 1 | 0 | 1 |
| R1 to curly-haired | 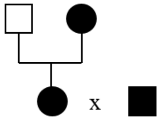 | [Mangalitza x Angeln Saddleback] x Mangalitza | 2 | 0 | 2 |
| | | [Mangalitza x European Wild Boar] x Mangalitza | 10 | 0 | 10 |
| R2 to straight-haired | 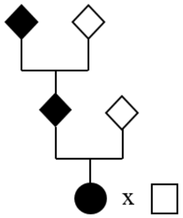 | [(Mangalitza x European Wild Boar) x Duroc] x Pietrain | 3 | 1 | 2 |

**Table S7** Results of filter analysis in WGS data for curly hair associated variants. The dataset incorporated 69 individuals (ten Mangalitza, three Mangalitza-crossbreeds and 56 straight haired controls from 25 distinct pig breeds and populations).

**Table S8** Minor allele frequency of 12 candidate variants for curly hair in Mangalitza in a total of 85 samples (n).

| Breed/population | Phenotype | n | NC_010443.5:g.25231855 G>A | NC_010443.5:g.236547263 C>T | NC_010444.4:g.61866070 T>C | NC_010446.5:g.66821473 C>T | NC_010448.4:g98006479 C>T | NC_010454.4:g.21253953 C>G | NC_010454.4:g.21358712 C>A | NC_010455.5:g.22932240 C>T | NC_010455.5:g.53728542 T>C | NC_010455.5:g.106612073 C>G | NC_010455.5:g.207222334 G>C | NC_010458.4:g.68450902 G>A |
|---|---|---|---|---|---|---|---|---|---|---|---|---|---|---|
| Gene | | | *TXLNB* | *FAM221B* | *CYP4F3* | *PREX2* | *APCDD1* | *KRT33A* | *KRTAP4-7* | *DLEC1* | *RYBP* | *SERPINI1* | *TRPM2* | *LARP1* |
| Minor allele | | | A | T | C | T | T | G | A | T | C | G | C | A |
| Mangalitza | curly-haired | 23 | 0.41 | 0.37 | 0.96 | 0.59 | 0.15 | 0.57 | 0.43 | 0.70 | 0.85 | 0.70 | 0.37 | 0.39 |
| Mangalitza x Angeln Saddleback | curly-haired | 12 | 0.46 | 0.50 | 0.50 | 0.29 | 0.25 | 0.83 | 0.79 | 0.29 | 0.29 | 0.50 | 0.17 | 0.38 |
| (Mangalitza x Angeln Saddleback) x Mangalitza | curly-haired | 2 | 0.50 | 0.50 | 0.75 | 0.26 | 0.00 | 0.00 | 0.00 | 0.75 | 0.75 | 0.75 | 0.00 | 0.00 |
| Angeln Saddleback | straight-haired | 3 | 0.00 | 0.00 | 0.00 | 0.00 | 0.00 | **0.17** | **0.17** | **0.17** | 0.00 | 0.00 | 0.00 | 0.00 |
| Angeln Saddleback x Husum Red Pied | straight-haired | 3 | 0.00 | **0.50** | 0.00 | **0.33** | **0.33** | 0.00 | 0.00 | 0.00 | 0.00 | 0.00 | 0.00 | 0.00 |
| European wild boar | straight-haired | 3 | 0.00 | 0.00 | 0.00 | 0.00 | 0.00 | 0.00 | 0.00 | **0.50** | 0.00 | 0.00 | 0.00 | 0.00 |
| Miniaturepig | straight-haired | 1 | 0.00 | 0.00 | 0.00 | 0.00 | 0.00 | 0.00 | 0.00 | 0.00 | 0.00 | 0.00 | 0.00 | 0.00 |
| German Landrace x German Large White | straight-haired | 3 | 0.00 | 0.00 | 0.00 | 0.00 | 0.00 | **0.17** | 0.00 | 0.00 | 0.00 | 0.00 | 0.00 | 0.00 |
| German Landrace x German Large White x Pietrain | straight-haired | 1 | 0.00 | 0.00 | 0.00 | 0.00 | 0.00 | 0.00 | 0.00 | 0.00 | 0.00 | 0.00 | 0.00 | 0.00 |

| | | | | | | | | | | | | | | |
|---|---|---|---|---|---|---|---|---|---|---|---|---|---|---|
| Husum Red Pied | straight-haired | 15 | 0.00 | **0.80** | 0.00 | **0.27** | **0.10** | **0.03** | 0.00 | 0.00 | 0.00 | 0.00 | 0.00 | 0.00 |
| Husum Red Pied x Bentheim Black Pied | straight-haired | 7 | 0.00 | **0.42** | 0.00 | **0.08** | **0.17** | **0.14** | 0.00 | 0.00 | 0.00 | 0.00 | 0.00 | 0.00 |
| Kune Kune | straight-haired | 3 | 0.00 | 0.00 | 0.00 | 0.00 | 0.00 | 0.00 | 0.00 | 0.00 | 0.00 | 0.00 | 0.00 | 0.00 |
| Mini-Lewe | straight-haired | 7 | 0.00 | 0.00 | 0.00 | 0.00 | 0.00 | 0.00 | 0.00 | 0.00 | 0.00 | 0.00 | 0.00 | 0.00 |
| Pietrain | straight-haired | 1 | 0.00 | 0.00 | 0.00 | 0.00 | 0.00 | 0.00 | 0.00 | 0.00 | 0.00 | 0.00 | 0.00 | 0.00 |
| Pot-bellied pig | straight-haired | 1 | 0.00 | 0.00 | 0.00 | 0.00 | 0.00 | **0.50** | 0.00 | 0.00 | 0.00 | 0.00 | 0.00 | 0.00 |

**Table S9** Minor allele frequency of six candidate variants for curly hair in Mangalitza in a total of 581 samples (n).

| Breed/population | Phenotype | n | NC_010443.5: g.25231855 G>A | NC_010444.4: g.61866070 T>C | NC_010455.5: g.53728542 T>C | NC_010455.5: g.106612073 C>G | NC_010455.5: g.207222334 G>C | NC_010458.4: g.68450902 G>A |
|---|---|---|---|---|---|---|---|---|
| Gene | | | *TXLNB* | *CYP4F3* | *RYBP* | *SERPINI1* | *TRPM2* | *LARP1* |
| Minor allele (=curly hair-associated) | | | A | C | C | G | C | A |
| Mangalitza | curly-haired | 117 | 0.69 | 0.91 | 0.63 | 0.54 | 0.47 | 0.23 |
| Mangalitza x Angeln Saddleback | curly-haired | 12 | 0.46 | 0.50 | 0.17 | 0.50 | 0.17 | 0.36 |
| Mangalitza x European Wild Boar | curly-haired | 6 | 0.50 | 0.50 | 0.50 | 0.08 | 0.33 | 0.25 |
| Mangalitza x Miniature pig | curly-haired | 9 | 0.50 | 0.50 | 0.22 | 0.50 | 0.22 | 0.17 |
| Mangalitza x Spotted Bentheim pig | curly-haired | 19 | 0.40 | 0.47 | 0.47 | 0.50 | 0.13 | 0.00 |
| (Mangalitza x Angeln Saddleback) x Mangalitza | curly-haired | 2 | 0.50 | 0.75 | 0.75 | 0.75 | 0.00 | 0.00 |

| | | | | | | | | |
|---|---|---|---|---|---|---|---|---|
| (Mangalitza x European Wild Boar) x Mangalitza | curly-haired | 10 | 0.75 | 0.35 | 0.30 | 0.30 | 0.20 | 0.05 |
| (Mangalitza x European Wild Boar) x (Mangalitza x European Wild Boar) | curly-haired | 1 | 0.50 | 0.50 | 0.50 | 0.50 | 0.00 | 0.5 |
| Mangalitza x European Wild Boar x Duroc x Pietrain | curly-haired | 2 | 0.00 | 0.00 | 0.25 | 0.25 | 0.00 | 0.00 |
| Mangalitza x European Wild Boar x Duroc x Pietrain | straight-haired | 1 | 0.00 | 0.00 | 0.00 | 0.00 | 0.00 | 0.00 |
| Angeln Saddleback | straight-haired | 3 | 0.00 | 0.00 | 0.00 | 0.00 | 0.00 | 0.00 |
| Angeln Saddleback x Husum Red Pied | straight-haired | 3 | 0.00 | 0.00 | 0.00 | 0.00 | 0.00 | 0.00 |
| European Wild Boar | straight-haired | 67 | 0.00 | 0.00 | **0.02** | 0.00 | 0.00 | 0.00 |
| German Landrace | straight-haired | 107 | 0.00 | 0.00 | 0.00 | 0.00 | 0.00 | 0.00 |

| | | | | | | | | |
|---|---|---|---|---|---|---|---|---|
| German Large White | straight-haired | 108 | 0.00 | 0.00 | 0.00 | 0.00 | 0.00 | 0.00 |
| German Landrace x German Large White | straight-haired | 3 | 0.00 | 0.00 | 0.00 | 0.00 | 0.00 | 0.00 |
| German Landrace x German Large White x Pietrain | straight-haired | 2 | 0.00 | 0.00 | 0.00 | 0.00 | 0.00 | 0.00 |
| German Landrace x Yorkshire x Pietrain | straight-haired | 4 | 0.00 | 0.00 | 0.00 | 0.00 | 0.00 | 0.00 |
| Goettingen Minipig | straight-haired | 18 | 0.00 | 0.00 | 0.00 | 0.00 | 0.00 | 0.00 |
| Miniature pig | straight-haired | 1 | 0.00 | 0.00 | 0.00 | 0.00 | 0.00 | 0.00 |
| Husum Red Pied | straight-haired | 17 | 0.00 | 0.00 | 0.00 | 0.00 | 0.00 | 0.00 |
| Husum Red Pied x Bentheim Black Pied | straight-haired | 7 | 0.00 | 0.00 | 0.00 | 0.00 | 0.00 | 0.00 |
| Iberico | straight-haired | 1 | 0.00 | 0.00 | 0.00 | 0.00 | 0.00 | 0.00 |

| | | | | | | | | |
|---|---|---|---|---|---|---|---|---|
| Kune-Kune | straight-haired | 3 | 0.00 | 0.00 | 0.00 | 0.00 | 0.00 | 0.00 |
| Leicoma | straight-haired | 30 | 0.00 | 0.00 | 0.00 | 0.00 | 0.00 | 0.00 |
| Meishan | straight-haired | 1 | 0.00 | 0.00 | 0.00 | 0.00 | 0.00 | 0.00 |
| Mini-Lewe | straight-haired | 11 | 0.00 | 0.00 | 0.00 | 0.00 | 0.00 | 0.00 |
| Pietrain | straight-haired | 2 | 0.00 | 0.00 | 0.00 | 0.00 | 0.00 | 0.00 |
| Pot-bellied pig | straight-haired | 1 | 0.00 | 0.00 | 0.00 | 0.00 | 0.00 | 0.00 |
| Bentheim Black Pied | straight-haired | 13 | 0.00 | 0.00 | 0.00 | 0.00 | 0.00 | 0.00 |

**Table S10** Error rate for genotypes inferred from imputation tested against KASP genotyping results. Error rate was calculated as frequency of incorrect genotypes (printed in bold) per individual and SNP.

| **Gene** | **SCA** | **Position** | **Genotype KASP** | **Genotype imputed** | **n** | **Error rate** |
|---|---|---|---|---|---|---|
| *TXLNB* | 1 | g.25231855G>A | AA | AA | 11 | |
| | | | AA | AG | **1** | |
| | | | AG | AA | **1** | |
| | | | AG | AG | 18 | |
| | | | GG | AG | **2** | |
| | | | GG | GG | 21 | |
| | | | AA | AA | 11 | 0.07 |
| *CYP4F3* | 2 | g.61866070T>C | CC | CC | 13 | |
| | | | CT | CT | 21 | |
| | | | TT | TT | 20 | 0.00 |
| *SERPINI1* | 13 | g.106612073C>G | CC | CC | 20 | |
| | | | CC | GC | **5** | |
| | | | GC | GC | 23 | |
| | | | GG | GG | 6 | 0.09 |
| *TRPM2* | 13 | g.207222334G>C | CC | CC | 2 | |
| | | | CC | CG | **1** | |
| | | | CG | GG | **3** | |
| | | | CG | CG | 3 | |
| | | | CG | GG | **10** | |
| | | | GG | GG | 35 | 0.26 |
| *LARP1* | 16 | g.68450902G>A | AG | AA | **2** | |
| | | | AG | AG | 12 | |
| | | | AG | GG | **2** | |
| | | | GG | AG | **4** | |
| | | | GG | GG | 34 | 0.15 |
| Mean error rate | | | | | | 0.11 |

**Table S11** Display of samples used for whole genome sequencing, SNP chip genotyping and ROH analysis in this study.

**Table S12** Primer sequences and PCR conditions used for validation of 12 candidate SNPs. Primer pairs, amplicon size (AS) in base pairs (bp), annealing (AT) and number of cycles, restriction enzyme and the corresponding incubation temperature (IT) are presented. Primers marked with FAM and HEX represent KASP-assays.

| Gene | Polymorphism | Forward primer(s) | Reverse primer (5'-3') | AS (bp) | AT (°C) /cycles | Restriction enzyme | IT (°C) |
|---|---|---|---|---|---|---|---|
| *TXLNB* | NC_010443.5: g.25231855G>A | 3'-GTTCCCTGGAGGTGTTGACTC-5'-FAM<br>GTGTTCCCTGGAGGTGTTGACTT-HEX | ACTAGTCAGTCTGCACAGCTGTCT | - | 61/38 | - | - |
| *FAM221B* | NC_010443.5: g.236547263C>T | 3'GGGCCTTCTCACTTCCCAGG-FAM<br>AGGGCCTTCTCACTTCCCAGA-HEX | GACCACTGGGTGGACGGCATAA | - | 61/29 | - | - |
| *CYP4F3* | NC_010444.4: g.61866070T>C | GGTCTTCTCCTCCTGATCCA | CAAGCCCATGTGAGAGTCTG | 528 | 58/42 | HpyCH4V | 37 |
| *PREX2* | NC_010446.5: g.66821473C>T | CCGAATCTTCCTGCTCATCTCC-FAM<br>CCCGAATCTTCCTGCTCATCTCT-HEX | AGAAACAGCAAACGCGTCTGTTTTAACAT | - | 61/35 | - | - |
| *APCDD1* | NC_010448.4: g98006479C>T | GTTCTACTACGGCGGCAACCG-FAM<br>AGTTCTACTACGGCGGCAACCA-HEX | GCCGCGGACCACCAGCGTA | - | 68/38 | - | - |
| *KRT33A* | NC_010454.4: g.21253953C>G | GCTAGTCACTGAATCCAGAACC | GACACCACCTGCTTGTTCAG | 444 | 58/42 | EcoO109I | 37 |
| *KRTAP4-7* | NC_010454.4: g.21358712C>A | GCAACAAGACACCCAAACTC | GGCGATAGTAAGTGGTGTGG | 525 | 58/42 | BsrI | 65 |
| *DLEC1* | NC_010455.5: g.22932240C>T | GAAGCCCAACCTGCGGCC-FAM<br>GGAAGCCCAACCTGCGGCT-HEX | GTACGTGATGCTGTCCATGGWGTA | - | 61/29 | - | - |
| *RYBP* | NC_010455.5: g.53728542T>C | CAGTATACAGTCAGCAAATGCCACAA-FAM<br>AGTATACAGTCAGCAAATGCCACAG-HEX | TTGAAGTGTGATTCGTTTCACTGGTCTTT | - | 61/29 | - | - |
| *SERPINI1* | NC_010455.5: g.106612073C>G | ACAGGAAGTTCCACTGGCCAC-FAM<br>ACAGGAAGTTCCACTGGCCAG-HEX | TTCTTCAAKGAGCTGTGCTTTGACCAA | - | 61/35 | - | - |
| *TRPM2* | NC_010455.5: g.207222334G>C | CCCTACGCCTTCCTGAGCG-FAM<br>CCCTACGCCTTCCTGAGCC-HEX | GGCATCTCCTGGACRTGCTTCTT | - | 61/29 | - | - |

| | | | | | | | |
|---|---|---|---|---|---|---|---|
| *LARP1* | NC_010458.4: g.68450902G>A | CGCCCGCGGCTCCCG-FAM<br>GCGCCCGCGGCTCCCA-HEX | GGCGCTGGCGGAGGAGCT | - | 61/29 | - | - |

**Table S13** Primer and reporter sequences used for validation of structural variant on SCA13 by five TaqMan Copy Number assays.

| **Assay ID** | **SCA** | **Target region** | **Forward primer sequence** | **Reverse primer sequence** | **Reporter (TaqMan MGB Probe)** |
|---|---|---|---|---|---|
| Control | 15 | *GCG* 68803685-68803814 | 5'-AACATTGCCAAACGTCACGATG-3' | 5'-GCCTTCCTCGGCCTTTCA-3' | 5'VIC-ACATGCTGAAGGGACC-3' |
| 13_42345_ CDGZFTM | 13 | *ENSSSCG 00000042345* 143209100-143209198 | 5'-GCCAGCAAAACCCACTCATCT-3' | 5'-CTCTCCCCACAGAAGGTTTGT-3' | 5'FAM-CCACAGTAAAAAAAAACAAAAACC-3' |
| 13_47219_ CDNKR3C | 13 | *ENSSSCG 00000047219* 143407478-143407587 | 5'-GTTATTCAGGATAAGGATCATATATGCTTTGAGT-3' | 5'-TTCCTTTTTGTAGGCTGCTATTTTGTTTT-3' | 5'FAM-CTTCTTCTACAGCCAGACTTTG-3' |
| 41302_ CDWCWX3 | 13 | *ENSSSCG 00000041302* 143330972-143331079 | 5'-GGTTGGCAGATAAAGAAAAAAGGAAGAA-3' | 5'-CGATTCAAATAAAGATGTTTTCCCTCTTACC-3' | 5'FAM-CTTCCTCCCACCCACCATCC-3' |
| End_Dup_ CDTZ9T9 | 13 | *ENSSSCG 00000045503* 143456903-143457010 | 5'-TGCATACAGTGACAGTTTGATCTCTT-3' | 5'-CTGCTCTTCAACATCGTTTTGGAA-3' | 5'FAM-TAGCCACAGCAATTAGACAAACAA-3' |
| Beg_Dup_ CDMFXHJ | 13 | *ENSSSCG 00000042345* 143207227-143207330 | 5'-GCGAGGGTCTTGATCATGAATGAAT-3' | 5'-CATTCCAAAGTAAGAGACCCAGTCA-3' | 5'FAM-TCAAATGCTTTTTCTGCGTCTATTG-3' |

**Table S14** Primer sequences and PCR conditions used for validation of translocation resulting in a gene fusion on SCA13. Primer pairs, amplicon size (AS) in base pairs (bp) for predicted and validated Breakpoint (BP), annealing temperature (AT) and number of cycles are presented.

| **Target region** | **Forward primer sequence** | **Forward primer position** | **Reverse primer sequence** | **Reverse primer position** | **AS (bp)** | **AT (°C) /cycles** |
|---|---|---|---|---|---|---|
| Wildtype allele: *ENSSSCG00000045503* | 5´ -GTGGTGAGAGTGGGCATCAG-3` (Primer F) | 13: 143457010-143457029 | 3´ -ATTCCAAAACCAGGCAGAGAT-5´ (Primer R1) | 13: 143457445-143457465 | 456 | 62°C/39 |
| Mutant allele : Gene Fusion of *ENSSSCG00000042345* with *ENSSSCG00000045503* | | | 3´ -TAGGACCAAGGAGAGGAAGGG-5´ (Primer R2) | 13: 143207339-143207359 | 274 (validated BP at SCA13: 143,207,203)<br><br>368 (predicted BP at SCA13: 143,207,109) | 62°C/39 |

## Figures

**Fig. S1** Graphic display of genomic region of the structural variant detected by LUMPY. (a) Wild type allele in the region of the two detected breakpoints on SCA13 at 143,207,203 bp and 143,457,136 bp. Positions of CNV assays are marked by stars, primers are represented by bars. (b) Mutant allele according to experimental data. A translocation of a fragment of ENSSSCG00000042345 (dark grey) results in a gene fusion with ENSSSCG00000045503 and a resulting modified open reading frame.

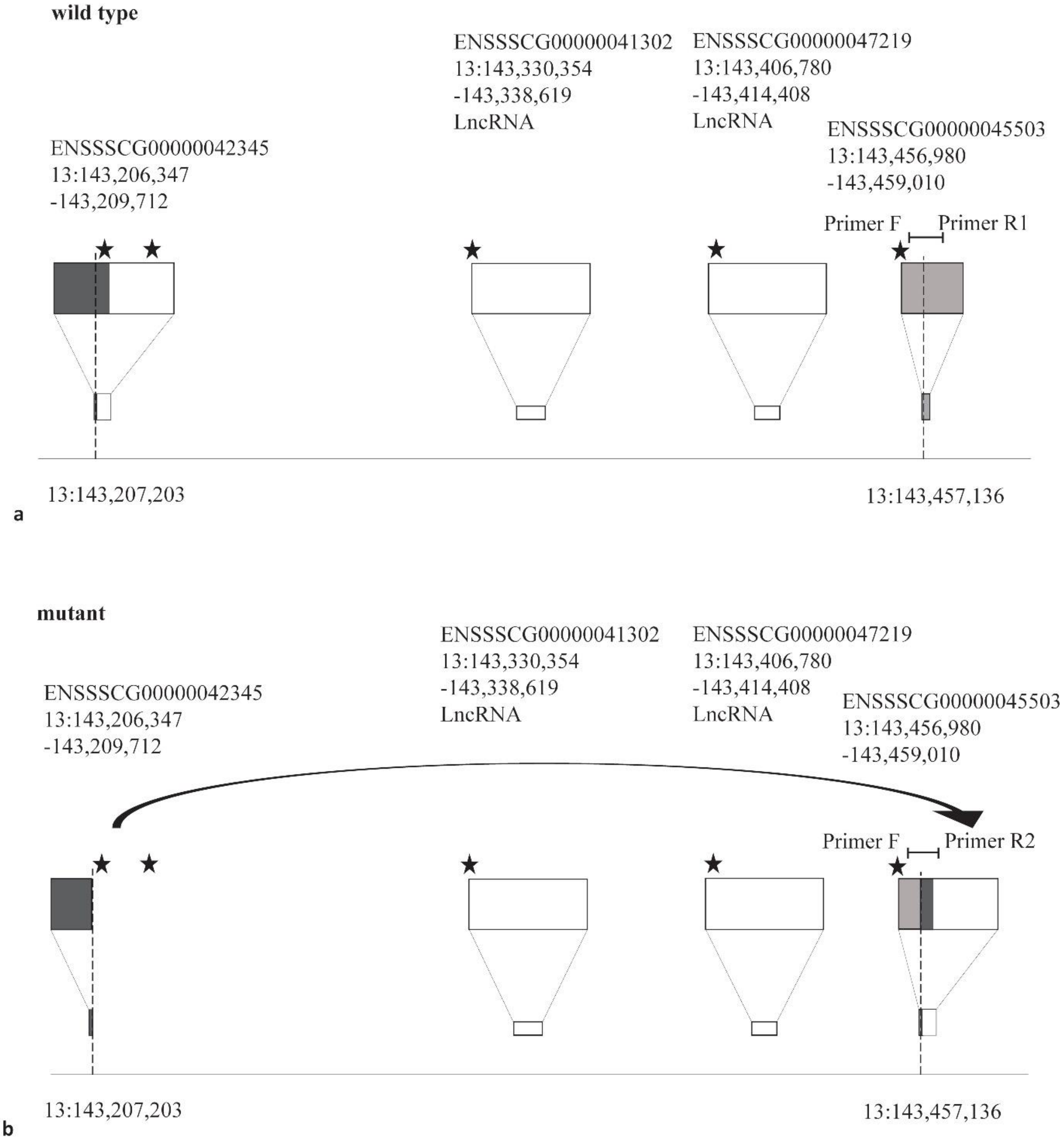

Supplement: Supplementary file 1 — Supplementary Information 1. [file 41598_2020_79037_MOESM1_ESM.pdf]
